# Supplementary material for: Green energy and steel imports reduce Europe’s net-zero infrastructure needs
Source: Nat Commun. 2025 Jun 12;16:5302. doi: 10.1038/s41467-025-60652-1 (PMC12162880; doi:10.1038/s41467-025-60652-1)
Supplement: Supplementary file 1 — Supplementary Information [file 41467_2025_60652_MOESM1_ESM.pdf]

# Supplementary Information:
# Green energy and steel imports reduce Europe's net-zero infrastructure needs

Fabian Neumann[1], Johannes Hampp[2], Tom Brown[1]

*[1]Department of Digital Transformation in Energy Systems, Institute of Energy Technology, Technische Universität Berlin, Fakultät III, Einsteinufer 25 (TA 8), 10587 Berlin, Germany*

*[2]Potsdam Institute for Climate Impact Research (PIK), Member of the Leibniz Association, P.O. Box 60 12 03, 14412 Potsdam, Germany*

# Supplementary Notes

Supplementary Note 1 Mathematical Model Formulation . . . . . . . . . . . . . . . . . . . . . 4

# Supplementary Tables

Supplementary Table 1 Overview of scenarios . . . . . . . . . . . . . . . . . . . . . . . . . . . . 9

# Supplementary Figures

Supplementary Figure 1 Schematic overview of the import supply chains . . . . . . . . . . . 7
Supplementary Figure 2 Overview of supply and consumption options per carrier . . . . . . . 8
Supplementary Figure 3 Overview of lowest direct hydrogen import costs into Europe . . . . . 10
Supplementary Figure 4 Overview of spatially fixed demands, when steel and ammonia industry can relocate . . . . . . . . . . . . . . . . . . . . . . . . . . . . . 11
Supplementary Figure 5 Overview of exogenous final energy and non-energy demand totals . 11
Supplementary Figure 6 Relocation patterns of steel and ammonia production in scenario without imports . . . . . . . . . . . . . . . . . . . . . . . . . . . . . . . 12
Supplementary Figure 7 Calculated import cost supply curve for hydrogen by pipeline and by ship . . . . . . . . . . . . . . . . . . . . . . . . . . . . . . . . . . . . . 13
Supplementary Figure 8 Calculated import cost supply curve for methane and ammonia by ship . . . . . . . . . . . . . . . . . . . . . . . . . . . . . . . . . . . . . 14
Supplementary Figure 9 Calculated import cost supply curve for methanol and Fischer-Tropsch by ship . . . . . . . . . . . . . . . . . . . . . . . . . . . . . . . . . . 15
Supplementary Figure 10 Calculated import cost supply curve for HBI and steel by ship . . . . 16
Supplementary Figure 11 Levelised cost supply curve of electricity in selected exporting regions 17
Supplementary Figure 12 Impact of technology development assumptions on total energy system cost with and without imports . . . . . . . . . . . . . . . . . . . 18
Supplementary Figure 13 Potential for cost reductions with reduced sets of import options for higher import costs . . . . . . . . . . . . . . . . . . . . . . . . . . . . 19
Supplementary Figure 14 Potential for cost reductions with reduced sets of import options for lower import costs . . . . . . . . . . . . . . . . . . . . . . . . . . . . 20
Supplementary Figure 15 Sensitivity of import volume on total system cost and composition for varying import costs . . . . . . . . . . . . . . . . . . . . . . . . . . 21
Supplementary Figure 16 Sensitivity of import volume on total system cost and composition for varying import costs . . . . . . . . . . . . . . . . . . . . . . . . . . 22
Supplementary Figure 17 Sensitivity of import volume on total system cost and composition for varying import costs . . . . . . . . . . . . . . . . . . . . . . . . . . 23
Supplementary Figure 18 Sensitivity of import volume on total system cost and composition for varying import costs . . . . . . . . . . . . . . . . . . . . . . . . . . 24
Supplementary Figure 19 Sensitivity of import volume on total system cost with subsets of import vectors available . . . . . . . . . . . . . . . . . . . . . . . . . . 25
Supplementary Figure 20 Sensitivity of import shares, mix and trade flows for import scenarios with restricted import vectors . . . . . . . . . . . . . . . . . . . 26
Supplementary Figure 21 Sensitivity of import shares, mix and trade flows for import scenarios with 10% lower costs . . . . . . . . . . . . . . . . . . . . . . . . 27
Supplementary Figure 22 Sensitivity of domestic cost supply curves for different import scenarios and carriers . . . . . . . . . . . . . . . . . . . . . . . . . . . . 28
Supplementary Figure 23 Sensitivity of temporal variations of domestic hydrogen and Fischer-Tropsch production costs for different import scenarios. . . . . . . . 29
Supplementary Figure 24 Sensitivity of energy balances for three import scenarios for the carriers electricity, heat, hydrogen and gas. . . . . . . . . . . . . . . . . 30
Supplementary Figure 25 Sensitivity of energy balances for three import scenarios for the carriers ammonia, methanol, and oil, as well as stored and atmospheric carbon dioxide . . . . . . . . . . . . . . . . . . . . . . . . . . . . . . . 31
Supplementary Figure 26 Sensitivity of layout of European energy infrastructure for different import scenarios . . . . . . . . . . . . . . . . . . . . . . . . . . . . . . 32

Supplementary Figure 27 Sensitivity of layout of European energy infrastructure for different import scenarios . . . 33
Supplementary Figure 28 Sensitivity of layout of European energy infrastructure for different import scenarios . . . 34
Supplementary Figure 29 Sensitivity of layout of European energy infrastructure for different import scenarios . . . 35
Supplementary Figure 30 Temporal usage pattern of electricity and hydrogen storage. . . . 36
Supplementary Figure 31 State-of-charge profile of long-duration energy storage in the domestic scenario without imports . . . 37
Supplementary Figure 32 Temporal usage pattern of backup power/heat in relation to import scenario . . . 38
Supplementary Figure 33 Spatial distribution of backup power for scenarios with all imports allowed and no imports . . . 39
Supplementary Figure 34 Temporal usage patterns of selected power-to-X technologies in scenario without imports. . . . 40
Supplementary Figure 35 Energy balance time series for electricity with and without imports . 41
Supplementary Figure 36 Energy balance time series for heat with and without imports . . . 42
Supplementary Figure 37 Energy balance time series for hydrogen with and without imports . 43
Supplementary Figure 38 Gas and electricity transmission network data . . . 44
Supplementary Figure 39 Locations considered for geological hydrogen storage in salt caverns 45
Supplementary Figure 40 Considered locations of industrial production sites by sector . . . 46

## Supplementary Note 1: Mathematical Model Formulation

This section describes the mathematical formulation of the energy system optimisation model PyPSA-Eur used in this study. This section is a reprint of a previous model description in Neumann et al. [14], which was updated and amended to explain how steel and ammonia industry relocation and the endogenous siting of hydrogen consumers for fuel synthesis and other applications are modelled. In the configuration used in this study, the model does not consider pathway optimisation (i.e. no sequences of investments), but searches for a cost-optimal layout corresponding to a given $CO_2$ emission reduction level and assumes perfect operational foresight for the weather year (2013) based on which capacities are optimised ('overnight scenario').

The objective is to minimise the total annual energy system costs of the energy system that comprises both investment costs and operational expenditures of generation, storage, transmission and conversion infrastructure. To express both as annual costs, we use the annuity factor $(1 - (1 + \tau)^{-n})/\tau$ that, like a mortgage, converts the upfront investment of an asset to annual payments considering its lifetime n and cost of capital $\tau$. Thus, the objective includes on one hand the annualised capital costs $c_*$ for investments at bus i in generator capacity $G_{i,r} \in \mathbb{R}^+$ of technology r (e.g. primary energy supply of gas, oil, nuclear, biomass), storage energy capacity $E_{i,s} \in \mathbb{R}^+$ of technology s (e.g. battery, heat, or hydrogen storage), electricity transmission line capacities $P_\ell \in \mathbb{R}^+$, and energy conversion (e.g. power-to-heat, electrolysis, Fischer-Tropsch synthesis) and transport (e.g. hydrogen/gas pipelines) capacities $F_k \in \mathbb{R}^+$ ('links'), as well as the variable operating costs $o_*$ for generator dispatch $g_{i,r,t} \in \mathbb{R}^+$ and link dispatch $f_{k,t} \in \mathbb{R}^+$ on the other:

$$\min_{G,E,P,F,g} \left[ \sum_{i,r} c_{i,r} \cdot G_{i,r} + \sum_{i,s} c_{i,s} \cdot E_{i,s} + \sum_{\ell} c_\ell \cdot P_\ell + \sum_k c_k \cdot F_k + \right. \tag{1}$$

$$\left. \sum_t w_t \cdot \left( \sum_{i,r} o_{i,r} \cdot g_{i,r,t} + \sum_k o_k \cdot f_{k,t} \right) \right]. \tag{2}$$

Thereby, the representative time snapshots t are weighted by the time span $w_t$ such that their total duration adds up to one year; $\sum_t w_t = 365 \cdot 24\text{h} = 8760\text{h}$. A bus i represents both a regional scope and an energy carrier. Represented carriers include electricity, heat (various subdivisions), hydrogen, ammonia, methane, methanol, oil, hot briquetted iron, steel, and carbon dioxide. Spatially resolved carriers are electricity, heat, hydrogen and methane (e.g. i ='region_id hydrogen'). For all other carriers, assuming negligible transport costs of the respective fuels, the model aggregates the regions to a single bus per carrier (e.g. i ='Europe methanol').

In addition to the cost-minimising objective function, we further impose a set of linear constraints that define limits on (i) the capacities of generation, storage, conversion and transmission infrastructure from geographical and technical potentials, (ii) the availability of variable renewable energy sources, performance of heat pumps and carrier-specific demands for each location and time step (iii) the limit for $CO_2$ emissions or transmission expansion, (iv) storage consistency equations, and (v) a multi-period linearised optimal power flow (LOPF) formulation and energy balance constraints for each region, carrier and time step. Overall, this results in a large linear problem (LP).

The capacities of generation, storage, conversion and transmission infrastructure are constrained from above by their installable potentials and from below by any existing components:

$$\underline{G}_{i,r} \leq G_{i,r} \leq \overline{G}_{i,r} \quad \forall i, r \tag{3}$$

$$\underline{E}_{i,s} \leq E_{i,s} \leq \overline{E}_{i,s} \quad \forall i, s \tag{4}$$

$$\underline{P}_\ell \leq P_\ell \leq \overline{P}_\ell \quad \forall \ell \tag{5}$$

$$\underline{F}_k \leq F_k \leq \overline{F}_k \quad \forall k \tag{6}$$

Moreover, the dispatch of generators and links may not only be constrained by their rated capacity but also by the weather-dependent availability of variable renewable energy or must-run conditions. This can be expressed as a time- and location-dependent availability factor $\overline{g}_{i,r,t}/\overline{f}_{k,t}$ and must-run factor $\underline{g}_{i,r,t}/\underline{f}_{k,t}$ (e.g. for certain power-to-X processes), given per unit of the nominal capacity:

$$\underline{g}_{i,r,t} G_{i,r} \leq g_{i,r,t} \leq \overline{g}_{i,r,t} G_{i,r} \quad \forall i, r, t \tag{7}$$

$$\underline{f}_{k,t} F_k \leq f_{k,t} \leq \overline{f}_{k,t} F_{i,r} \quad \forall k, t \tag{8}$$

The parameter $\underline{f}_{k,t}$ can also be used to define whether a link is bidirectional or unidirectional. For instance, for HVDC links $\underline{f}_{k,t} = -1$ would allow lossless power flows in either direction. On the other hand, a heat resistor has $\underline{f}_{k,t} = 0$ since it can only convert electricity to heat, not the other way around.

The energy levels $e_{i,s,t}$ of all stores are constrained by their energy capacity

$$0 \leq e_{i,s,t} \leq E_{i,s} \quad \forall i, s, t, \tag{9}$$

and have to be consistent with the dispatch variable $h_{i,s,t} \in \mathbb{R}$ in all hours

$$e_{i,s,t} = \eta_{i,s,0}^{w_t} \cdot e_{i,s,t-1} + w_t \cdot h_{i,s,t}, \tag{10}$$

where $\eta_{i,s,0}$ denotes the standing loss. Furthermore, the storage energy levels are either assumed to be cyclic or given an initial state of charge,

$$e_{i,s,0} = e_{i,s,T} \quad \forall i, s, \tag{11}$$

$$\text{or} \quad e_{i,s,0} = e_{i,s,\text{initial}} \quad \forall i, s. \tag{12}$$

The modelling of hydroelectricity storage deviates from regular storage to additionally account for natural inflow and spillage of water. We also assume fixed power ratings $H_{i,s}$ for hydroelectricity storage. The dispatch of hydroelectricity storage units is split into two positive variables; one each for charging $h^+_{i,s,t}$ and discharging $h^-_{i,s,t}$, and limited by $H_{i,s}$.

$$0 \leq h^+_{i,s,t} \leq H_{i,s} \quad \forall i, s, t \tag{13}$$

$$0 \leq h^-_{i,s,t} \leq H_{i,s} \quad \forall i, s, t \tag{14}$$

The energy levels $e_{i,s,t}$ of all hydroelectric storage also have to match the dispatch across all hours

$$\begin{aligned} e_{i,s,t} = \eta_{i,s,0}^{w_t} \cdot e_{i,s,t-1} + w_t \cdot h^{\text{inflow}}_{i,s,t} - w_t \cdot h^{\text{spillage}}_{i,s,t} \qquad \forall i, s, t \\ + \eta_{i,s,+} \cdot w_t \cdot h^+_{i,s,t} - \eta^{-1}_{i,s,-} \cdot w_t \cdot h^-_{i,s,t}, \end{aligned} \tag{15}$$

whereby hydropower storage units can additionally have a charging efficiency $\eta_{i,s,+}$, a discharging efficiency $\eta_{i,s,-}$, natural inflow $h^{\text{inflow}}_{i,s,t}$ and spillage $h^{\text{spillage}}_{i,s,t}$, besides the standing loss $\eta_{i,s,0}$.

The nodal balance constraint for supply and demand (Kirchoff's current law for electricity buses, or simply energy balances for all other carriers) requires local generators and storage units, incoming or outgoing energy flows $p_{\ell,t}$ of incident transmission lines $\ell$, and incoming or outgoing energy flows $f_{k,t}$ of pipelines and converters k (e.g. electrolysis, heat pumps, Fischer-Tropsch synthesis, hydrogen turbines, electric arc furnace) to balance the perfectly inelastic, exogenously given demand $d_{i,t}$ at each location i and snapshot t

$$\sum_r g_{i,r,t} + \sum_s \left( h^-_{i,s,t} - h^+_{i,s,t} \right) + \sum_s h_{i,s,t} + \sum_\ell K_{i\ell} p_{\ell,t} + \sum_k L_{ikt} f_{k,t} = d_{i,t} \quad \forall i, t, \tag{16}$$

where $K_{i\ell}$ is the incidence matrix of the electricity network with non-zero values −1 if line $\ell$ starts at node i and 1 if it ends at node i. $L_{ikt}$ is the pipeline and conversion incidence matrix of the network with non-zero values −1 if link k starts at node i and $\eta_{i,k,t}$ if one of its terminal buses is node i. For a link with more than two outputs (e.g. CHP converts gas to heat and electricity in a fixed ratio), the respective column of the conversion incidence matrix has more than two non-zero entries. These entries may also be negative to denote additional inputs rather than multiple outputs. For instance, modelling Fischer-Tropsch synthesis requires hydrogen and carbon dioxide as inputs to produce liquid hydrocarbons ('oil'). The operational decision variable $f_{k,t}$ is always associated with one input; additional inputs would be modelled as outputs with a negative entry for $\eta_{i,k,t}$. These entries may also contain unit conversions (e.g. for Fischer-Tropsch synthesis from carbon dioxide input in tonnes to oil output in MWh). Moreover, the factor $\eta_{i,k,t}$ can also be time-dependent and greater than one for certain technologies (e.g. for heat pumps converting electricity and ambient heat to hot water), which can result in additional primary energy input to the model scope (ambient heat).

By modelling the conversion between different energy carriers in this way, i.e. as links with capacity $F_k$, dispatch $f_{k,t}$ and conversion incidence matrix $L_{ikt}$, the model can endogenously decide some consumption

patterns at bus i in addition to the exogenously given demands $d_{i,t}$. Noting that bus i represents a region/carrier combination (e.g. 'region_id hydrogen' or 'Europe methanol'), k would conversely represent a region/technology combination (e.g. 'region_id electrolyser'). While the positive terms of $\sum_k L_{ikt} f_{k,t}$ in the nodal balance constraints, supplementary equation (16), represent endogenous supply at bus i, the negative terms represent endogenous consumption (e.g. consumption of electricity for electrolysis, or hydrogen for methanolisation in a region). What consumption and supply patterns are possible then depends on where the model decides to build capacity $F_k$ (e.g. of electrolysis in a region). For our relocation modelling of steel and ammonia production, we keep $F_k$ as free variables for Haber-Bosch, direct iron reduction and electric arc furnace links for each region, effectively allowing greenfield siting of these industries. In scenarios without relocation, we fix $F_k$ to the current regional capacities of these technologies such that no new sites for steel and ammonia production can be developed.

The effective power flows $p_{\ell,t}$ are limited by their nominal capacities $P_\ell$ minus losses $\psi_\ell$.

$$|p_{\ell,t}| \leq \bar{p}_\ell P_\ell - \psi_\ell \qquad \forall \ell, t, \tag{17}$$

where $\bar{p}_\ell$ acts as an additional per-unit security margin on the line capacity to allow a buffer for the failure of single circuits ($N-1$ criterion) and reactive power flows.

Kirchoff's voltage law (KVL) imposes further constraints on the flow of AC transmission lines and there are several ways to formulate KVL with large impacts on performance. Here, we use linearised load flow assumptions, where the voltage angle difference around every closed cycle in the electricity transmission network must add up to zero. Using a cycle basis $C_{\ell c}$ of the network graph where the independent cycles c are expressed as directed linear combinations of lines $\ell$, we can write KVL as

$$\sum_\ell C_{\ell c} \cdot x_\ell \cdot p_{\ell,t} = 0 \qquad \forall c, t \tag{18}$$

where $x_\ell$ is the series inductive reactance of line $\ell$.

The AC transmission losses $\psi_\ell$ are approximated as a tangent-based linear approximation of the loss parabola $\psi_\ell = r_\ell p_\ell^2$, where $r_\ell$ is the resistance, following Neumann et al. [98]:

$$0 \leq \psi_\ell \leq r_\ell (\bar{p}_\ell \bar{P}_\ell)^2 \qquad \forall \ell \tag{19}$$

$$\psi_\ell \geq m_k \cdot p_\ell + a_k \qquad \forall \ell, k = 1, \ldots, n \tag{20}$$

$$\psi_\ell \geq -m_k \cdot p_\ell + a_k \qquad \forall \ell, k = 1, \ldots, n \tag{21}$$

For each segment k of the total n segments, we derive the slope $m_k$ and offset $a_k$ in the following way:

$$\psi_\ell(k) = r_\ell \left( \frac{k}{n} \cdot \bar{p}_\ell \bar{P}_\ell \right)^2 \tag{22}$$

$$m_k = \frac{d\psi_\ell(k)}{dk} = 2 r_\ell \left( \frac{k}{n} \cdot \bar{p}_\ell \bar{P}_\ell \right) \tag{23}$$

$$a_k = \psi_\ell(k) - m_k \left( \frac{k}{n} \cdot \bar{p}_\ell \bar{P}_\ell \right). \tag{24}$$

The losses also extend the left-hand side of the nodal balance constraints in supplementary equation (16) by the term $-0.5 \cdot |K_{i\ell}| \cdot \psi_\ell$, splitting the losses equally between both connection points.

Finally, we add a constraint so that the total $CO_2$ emissions net out to zero over the year. The emissions are determined from the difference between final and initial 'filling level' of the store used to represent $CO_2$ in the atmosphere.

$$e_{CO_2,\text{atmosphere},t=T} - e_{CO_2,\text{atmosphere},t=0} \leq 0. \tag{25}$$

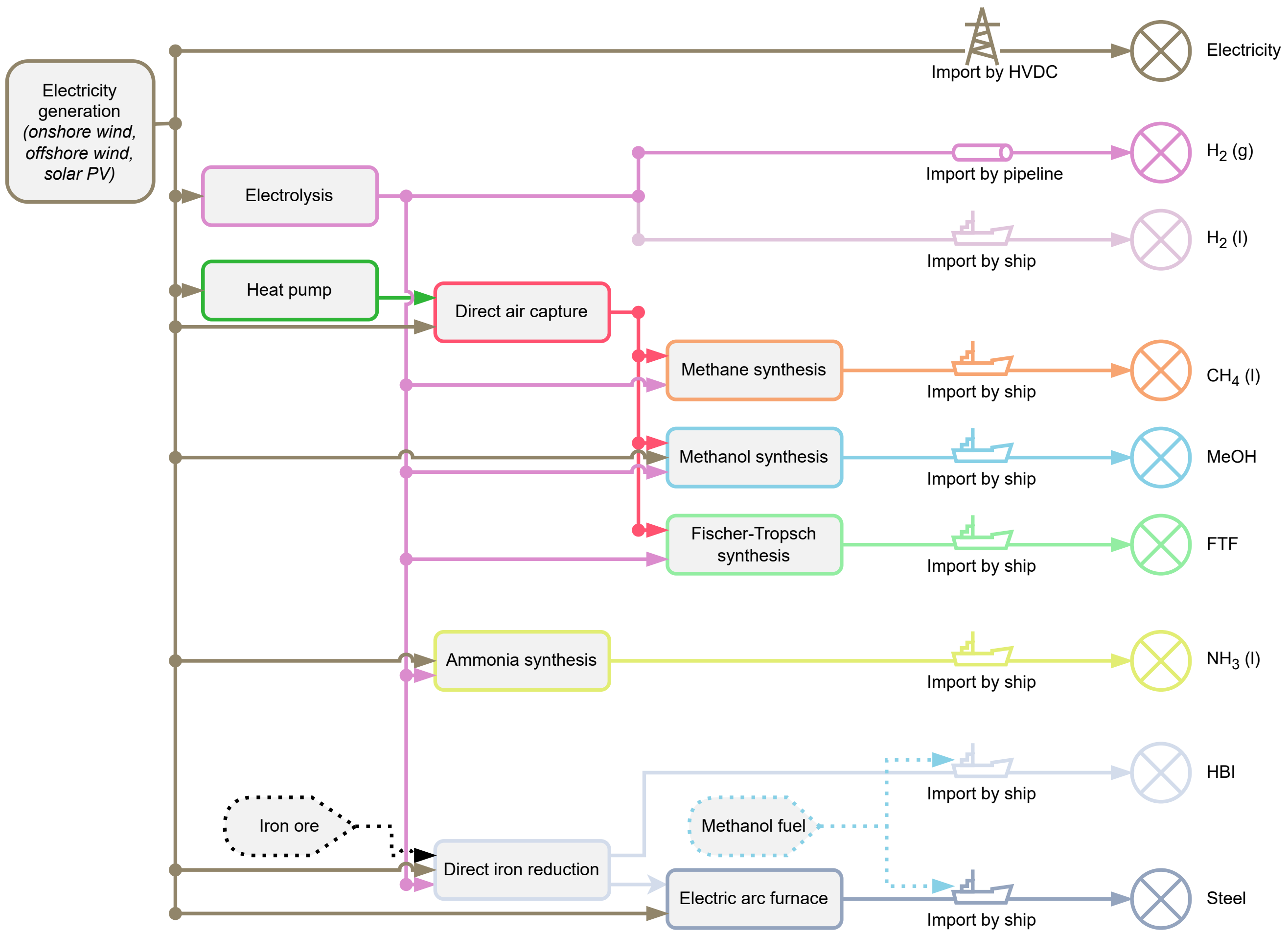


Supplementary Figure 1: **Schematic overview of the import supply chains.** The illustration includes key input-output ratios of the different conversion processes and the transport efficiencies for the different import vectors.

**Electricity (115 regions)**

| Supply | Withdrawal |
|---|---|
| rooftop solar | industry electricity |
| utility-scale solar | residential electricity |
| onshore wind | services electricity |
| offshore wind (fixed-pole/floating, AC/DC-connected) | agriculture electricity |
| nuclear | air-sourced heat pump |
| hydro reservoirs | ground-sourced heat pump |
| pumped-hydro | resistive heater |
| run-of-river | electric vehicle charger |
| import by HVDC link | battery charger |
| gas CHP (w/wo CC) | pumped-hydro |
| biomass CHP (w/wo CC) | hydrogen pipeline (compression) |
| gas turbine (OCGT) | direct air capture |
| methanol turbine (OCGT) | Haber-Bosch |
| hydrogen turbine (OCGT) | electric arc furnace |
| hydrogen fuel cell CHP | direct iron reduction |
| battery discharger | distribution grid losses |
| vehicle-to-grid | transmission grid losses |
| | methanolisation |
| | electrolysis |

**Grids & Storage**: distribution grid; transmission grid; battery storage; pumped-hydro storage; electric vehicles

**Hydrogen (115 regions)**

| Supply | Withdrawal |
|---|---|
| import by pipeline | Fischer-Tropsch |
| import by ship | methanolisation |
| electrolysis | electrobiofuels |
| chlor-alkali electrolysis (exogenous) | direct iron reduction |
| steam methane reforming (w/wo CC) | Haber-Bosch |
| ammonia cracker | hydrogen turbine (OCGT) |
| | hydrogen fuel cell CHP |
| | methanol-to-kerosene |
| | Sabatier |

**Grids & Storage**: new pipelines; retrofitted pipelines; storage in salt caverns; storage in steel tanks

**Liquid Hydrocarbons (not spatially resolved)**

| Supply | Withdrawal |
|---|---|
| import by ship | kerosene for aviation |
| fossil oil refining | naphtha for industry |
| Fischer-Tropsch | diesel for agriculture |
| electrobiofuels | |

**Storage**: hydrocarbon storage

**Methanol (not spatially resolved)**

| Supply | Withdrawal |
|---|---|
| import by ship | methanol turbine (OCGT) |
| methanolisation | methanol for shipping |
| | methanol for industry |
| | methanol-to-kerosene |

**Storage**: hydrocarbon storage

**Methane (not spatially resolved)**

| Supply | Withdrawal |
|---|---|
| import by ship | gas for high-T industry heat (w/wo CC) |
| fossil gas | steam methane reforming (w/wo CC) |
| biogas upgrading (w/wo CC) | gas boiler (rural/urban) |
| Sabatier | gas CHP |
| | gas turbine (OCGT) |

**Storage**: hydrocarbon storage

**Ammonia (not spatially resolved)**

| Supply | Withdrawal |
|---|---|
| import by ship | ammonia cracker |
| Haber-Bosch | ammonia for fertilizer |

**Storage**: ammonia tank

**Heat (115 regions)**

| Supply | Withdrawal |
|---|---|
| air-sourced heat pump | residential heat |
| ground-sourced heat pump (only rural) | services heat |
| resistive heater | agriculture heat |
| gas boiler | low-T industry heat |
| biomass boiler | direct air capture |
| solar thermal | water tank charger |
| water tank discharger | |
| biomass CHP (w/wo CC, only DH) | |
| gas CHP (w/wo CC, only DH) | |
| hydrogen fuel cell CHP (only DH) | |
| electrolysis (only DH) | |
| Haber-Bosch (only DH) | |
| Sabatier (only DH) | |
| Fischer-Tropsch (only DH) | |
| methanolisation (only DH) | |

**Storage**: long-duration thermal storage (only DH); hot water tank

**CO2 atmosphere (not spatially resolved)**

| Supply | Withdrawal |
|---|---|
| kerosene for aviation | solid biomass for industry (w CC) |
| diesel for agriculture | solid biomass CHP (w CC) |
| methanol for shipping | biogas upgrading (w CC) |
| methanol for industry | direct air capture |
| naphtha for industry | electrobiofuels |
| gas boiler | |
| gas CHP (w/wo CC) | |
| gas turbine (OCGT) | |
| methanol turbine (OCGT) | |
| process emissions (w/wo CC) | |
| fossil oil refining | |
| gas for high-T industry heat (w/wo CC) | |
| steam methane reforming (w/wo CC) | |

**CO2 commodity (not spatially resolved)**

| Supply | Withdrawal |
|---|---|
| direct air capture | Fischer-Tropsch |
| biogas upgrading (w CC) | methanolisation |
| gas CHP (w CC) | sequestration |
| biomass CHP (w CC) | Sabatier |
| steam methane reforming (w CC) | |
| process emissions (w CC) | |
| solid biomass for industry (w CC) | |
| gas for high-T industry heat (w CC) | |

**Storage**: intermediate storage in steel tank; long-term geological sequestration

Supplementary Figure 2: **Overview of supply and consumption options per carrier.** Each technology inherits the resolution of the highest resolved carrier it connects to (i.e. any technology that consumes electricity exists as investment option for each of the 115 regions). AC = alternating current; DC = direct current; OCGT = open cycle gas turbine; CHP = combined heat and power; CC = carbon capture; DH = district heating; high-T = high temperature.

Supplementary Table 1: **Overview of scenarios.** Overall, 352 scenario runs have been computed. The estimate for a single re-run of all scenarios is in the order of 50,000 CPU-hours. Figure numbers with the prefix 'S' denote supplementary figures. * Some combinations of import carriers and volumes have been omitted in case they exceeded the model's ability to absorb these.

| Scenario group | Import carriers | Import volume [TWh] | Import costs | Technology assumptions year | St. / $NH_3$ relocation | Figures | Runs |
|---|---|---|---|---|---|---|---|
| **Subsets of import carriers** | none, all, methane, electricity, ammonia, HBI/steel, hydrogen, methanol, FT, methanol/FT, only carbonaceous fuels, hydrogen derivatives, hydrogen derivatives and steel, all but electricity and steel, all but electricity | any | default (±0%) | 2030, 2040, 2050 | yes, no (for 2040) | Figs. 2, 3, 4, 7, Supplementary Figs. 6, 12, 20, 22, 23, 24, 25, 26, 28, 29ff. | 60 |
| **Subsets of import carriers (import cost variation)** | all, methane, electricity, ammonia, HBI/steel, hydrogen, methanol, FT, methanol/FT, only carbonaceous fuels, hydrogen derivatives, hydrogen derivatives and steel, all but electricity and steel, all but electricity | any | -20%, -10%, +10%, +20% | 2040 | yes | Supplementary Figs. 13, 14, 21, 27 | 56 |
| **Import cost variation (all import carriers)** | all | any | -50%, -40%, -30%, -20%, -10%, +10%, +20%, +30%, +50% | 2030, 2040, 2050 | yes | Figs. 5 | 27 |
| **Import cost variation (all import carriers but electricity)** | all | any | -50%, -40%, -30%, -20%, -10%, +10%, +20%, +30%, +50% | 2030, 2040, 2050 | yes | Figs. 5 | 27 |
| **Import cost variation (only carbonaceous import carriers)** | all | any | -50%, -40%, -30%, -20%, -10%, +10%, +20%, +30%, +50% | 2030, 2040, 2050 | yes | Figs. 5, Supplementary Figs. 27 | 27 |
| **Import cost variation (only electricity)** | all | any | -30%, -20%, -10% | 2040 | yes | – | 3 |
| **Import volumes (subsets of import carriers)** | all, electricity, hydrogen, methane, ammonia, methanol/FT, all but electricity | 500, 1000, 1500, 2000, 3000, 4000, 5000, 6000, 7000, 8000, 9000, 10000* | default (±0%) | 2040 | Yes | Figs. 6, Supplementary Figs. 19 | 47 |
| **Import volumes (import cost variation)** | all | 500, 1000, 1500, 2000, 3000, 4000, 5000, 6000, 7000, 8000, 9000, 10000 | -50%, -30%, -20%, -10%, +10%, +20%, +30%, +50% | 2040 | yes | Figs. 6, Supplementary Figs. 15, 16, 17, 18, | 96 |
| **Sensitivity: No hydrogen network** | none, all, all but hydrogen | any | default (±0%) | 2040 | yes | Supplementary Figs. 28 | 3 |
| **Sensitivity: no PtX waste heat** | none, all | any | default (±0%) | 2040 | yes | – | 2 |
| **Sensitivity: all PtX waste heat** | none, all | any | default (±0%) | 2040 | yes | Supplementary Figs. 26 | 2 |
| **Sensitivity: PtX flexibility** | none, all | any | default (±0%) | 2040 | yes | Supplementary Figs. 28 | 2 |

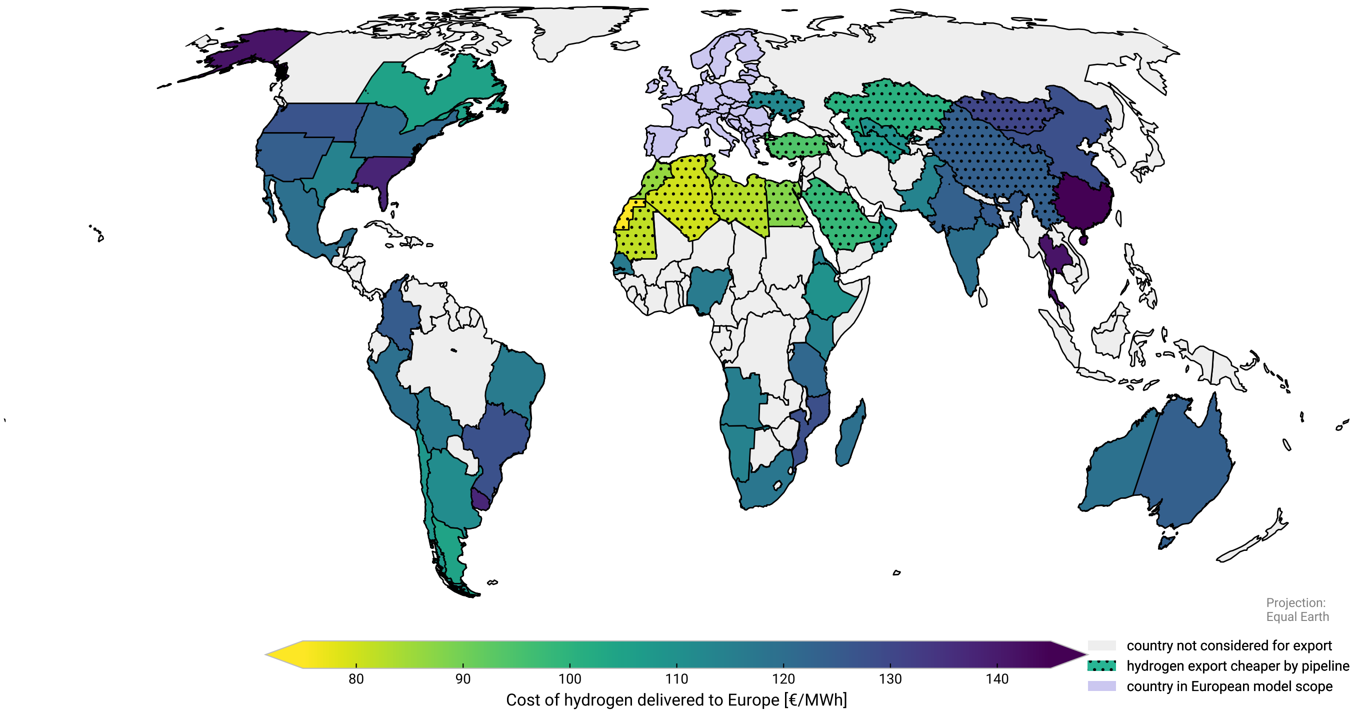


Supplementary Figure 3: **Overview of lowest direct hydrogen import costs into Europe per exporting country.** Supplement to Figure 1. Dotted areas indicate that hydrogen export from the respective region is cheaper by pipeline than by ship. Maps made with Natural Earth. Subnational regions created from geoBoundaries [135].

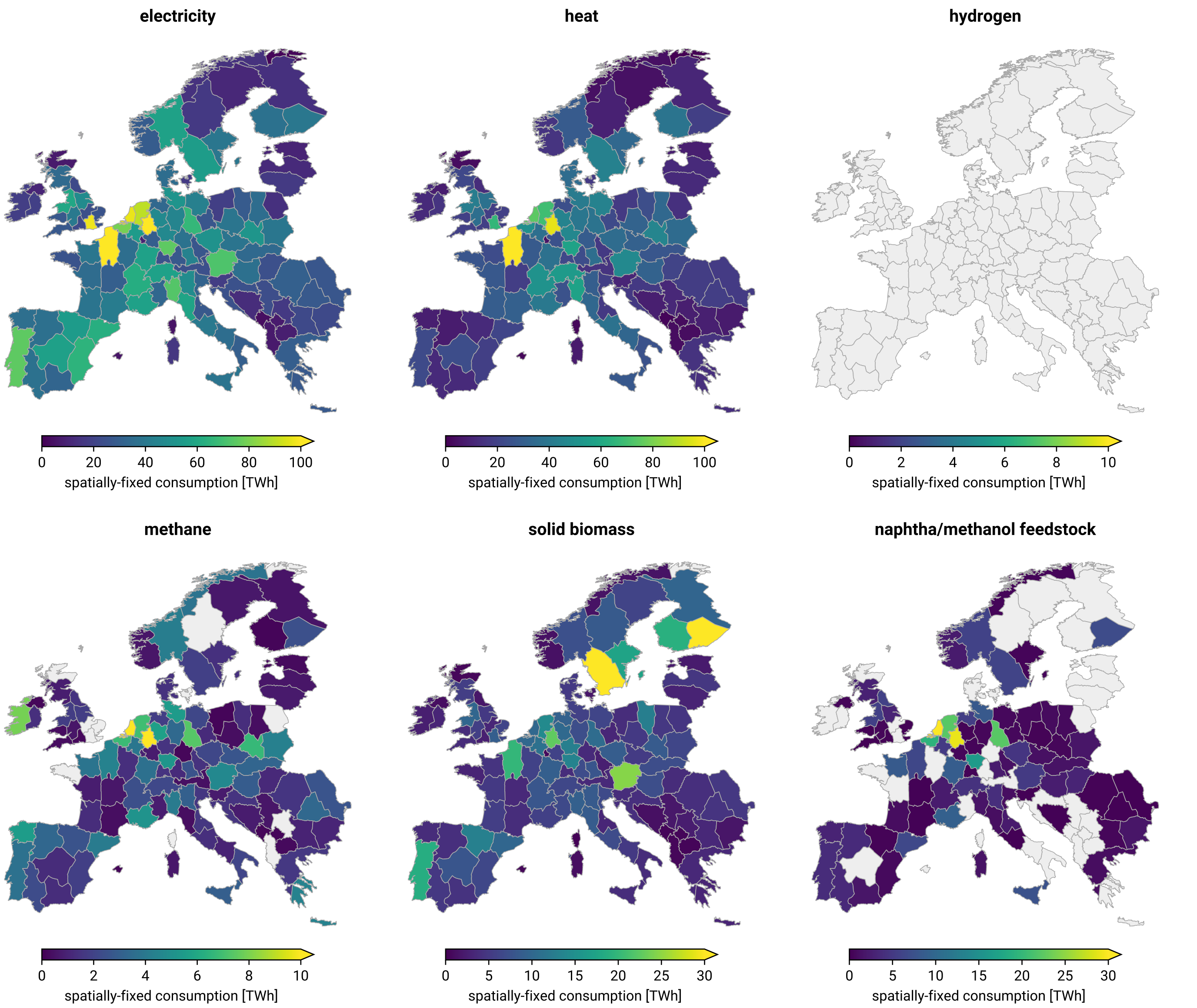


Supplementary Figure 4: **Overview of spatially fixed demands, when steel and ammonia industry can relocate.** In this scenario, there is virtually no spatially fixed hydrogen demand. Maps made with Natural Earth.

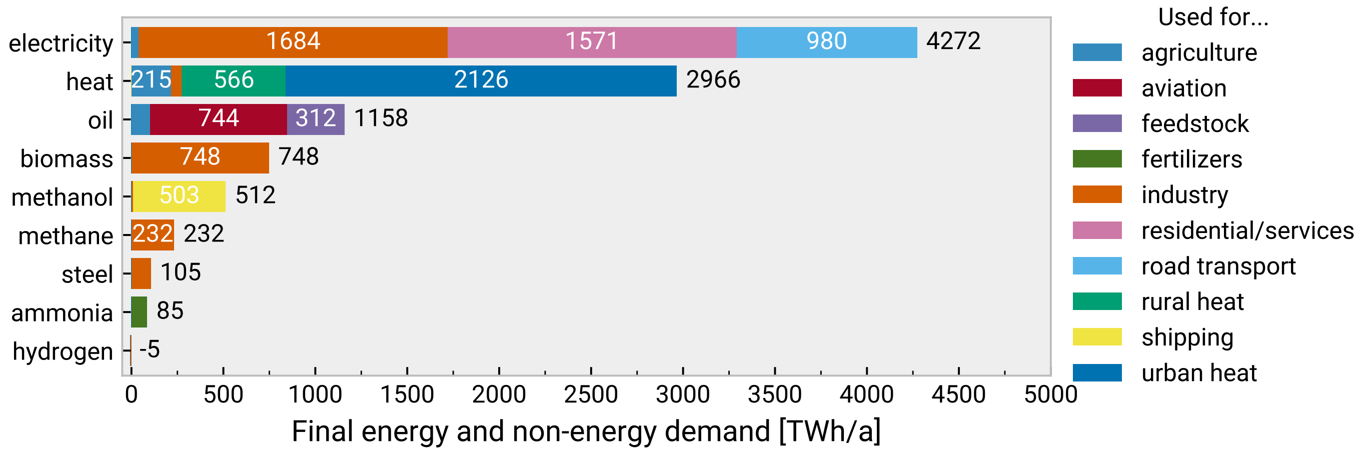


Supplementary Figure 5: **Overview of exogenous final energy and non-energy demand totals.** The energy content of steel is given as 2.1 MWh per tonne.

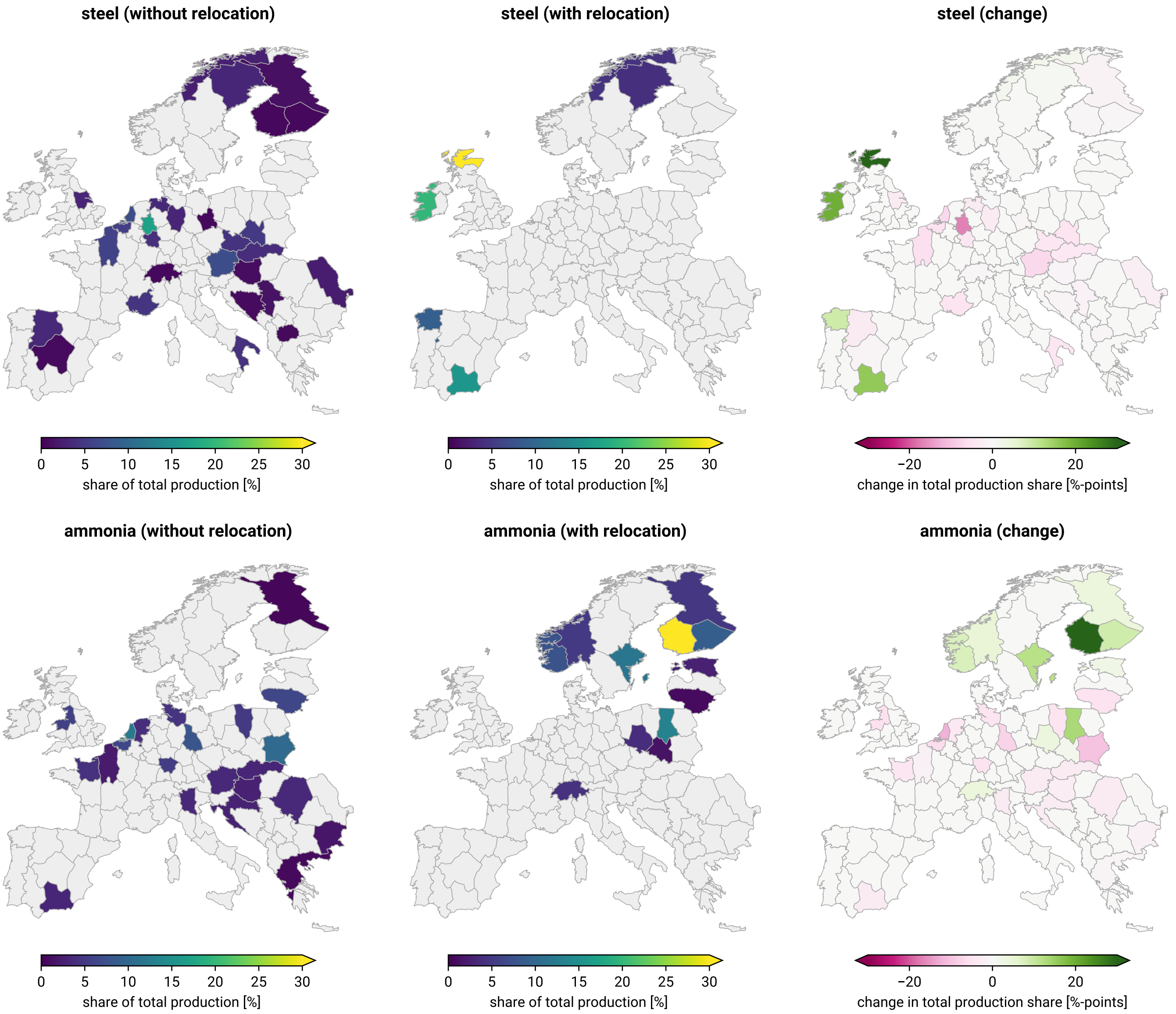


Supplementary Figure 6: **Relocation patterns of steel and ammonia production in scenario without imports.** All values are given relative to the total production volume. The left column shows the original regional distribution of steel and ammonia production volumes. The centre column shows the endogenously optimized allocation of steel and ammonia production. The right column shows the absolute change in the regions' total production share. Much of the steel production moves to Spain, Scotland and Ireland. A substantial share of ammonia production relocates to Finland. Maps made with Natural Earth.

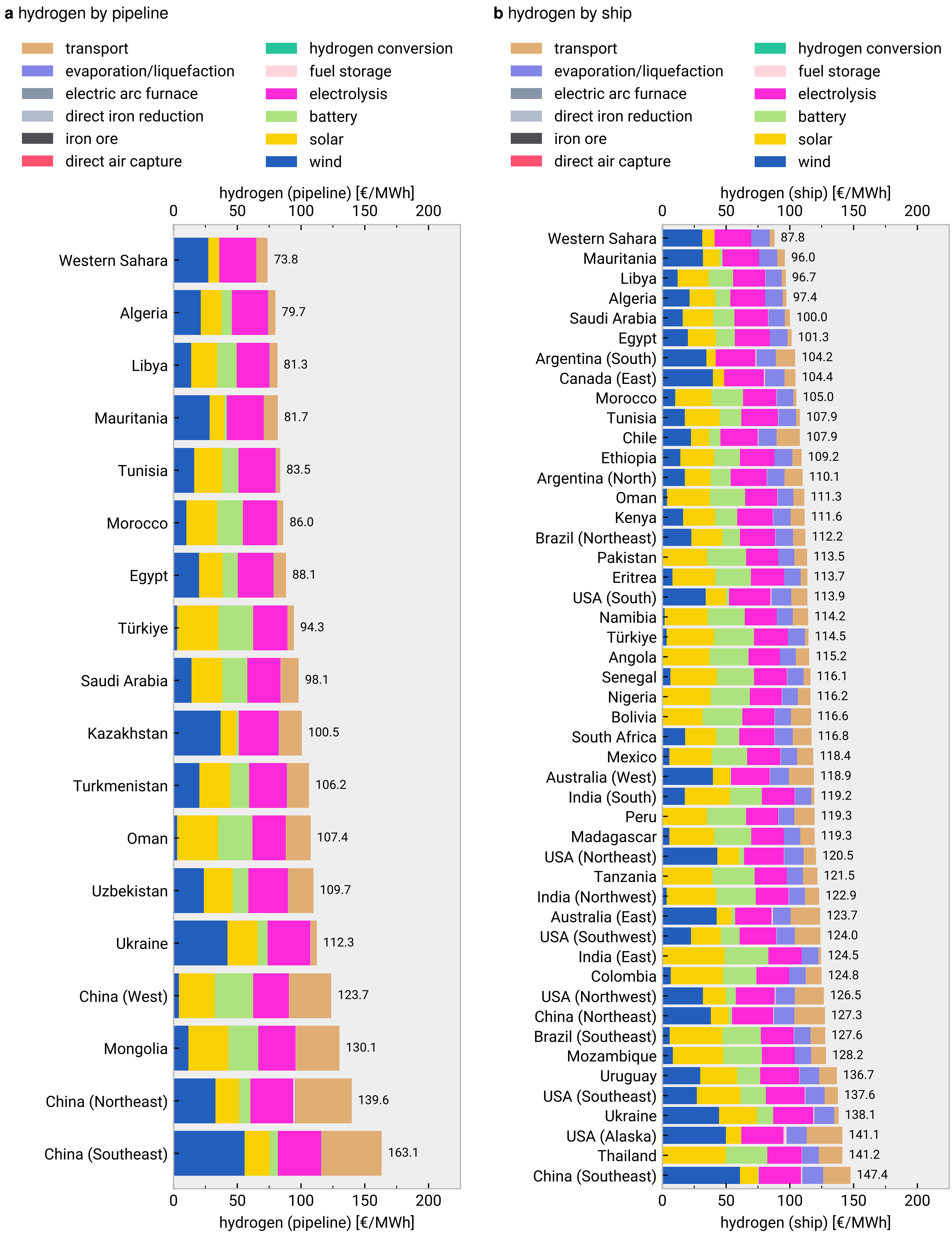


Supplementary Figure 7: **Calculated import cost supply curve for hydrogen by pipeline and by ship.** The levelised cost for each region have been calculated for an annual export volume of 500 TWh. Some landlocked regions in Central Asia have been excluded from ship-based exports. Some countries have been excluded from pipeline-based exports due to unrealistic distances to bridge.

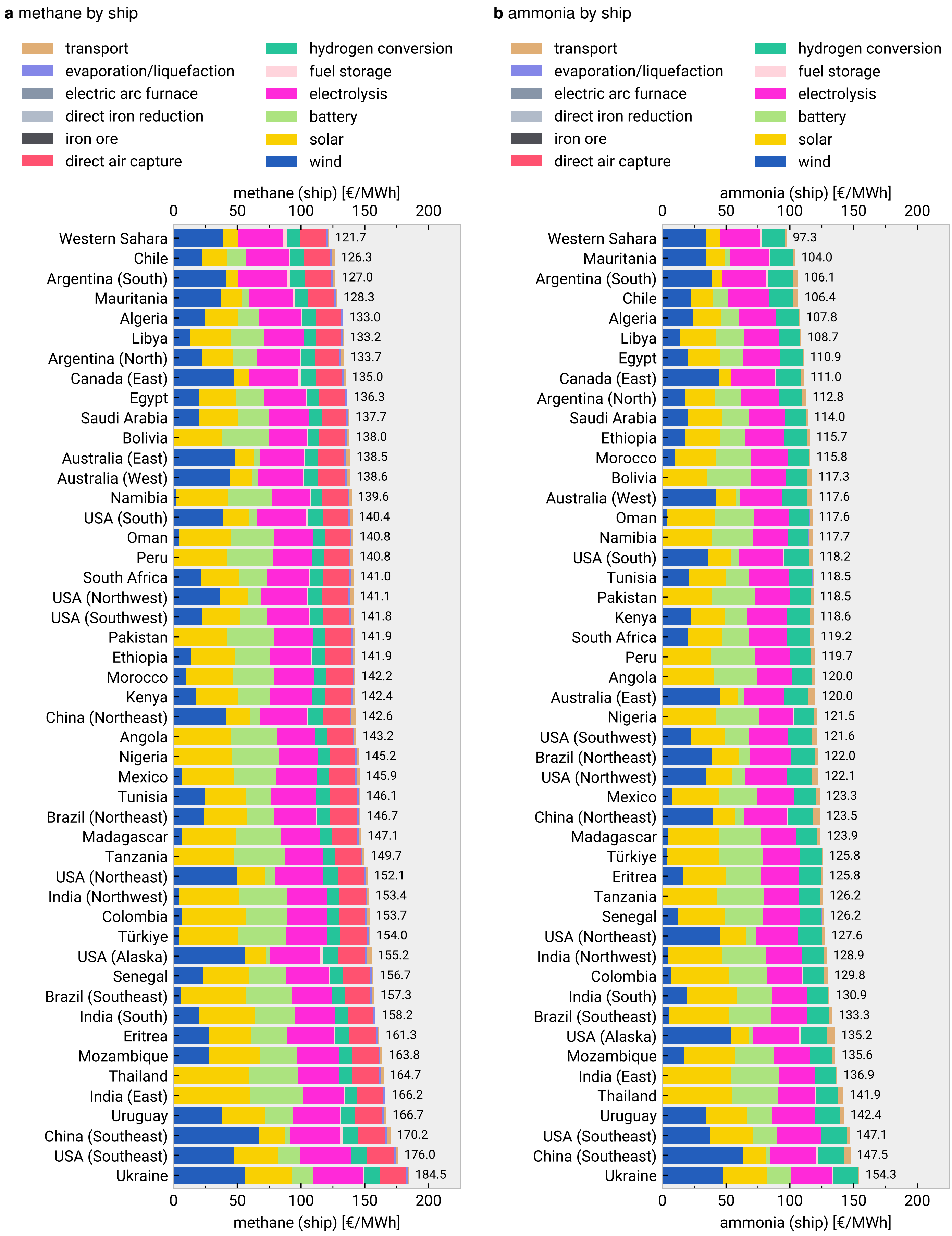


Supplementary Figure 8: **Calculated import cost supply curve for methane and ammonia by ship.** The levelised cost for each region have been calculated for an annual export volume of 500 TWh. Some landlocked regions in Central Asia have been excluded from ship-based exports.

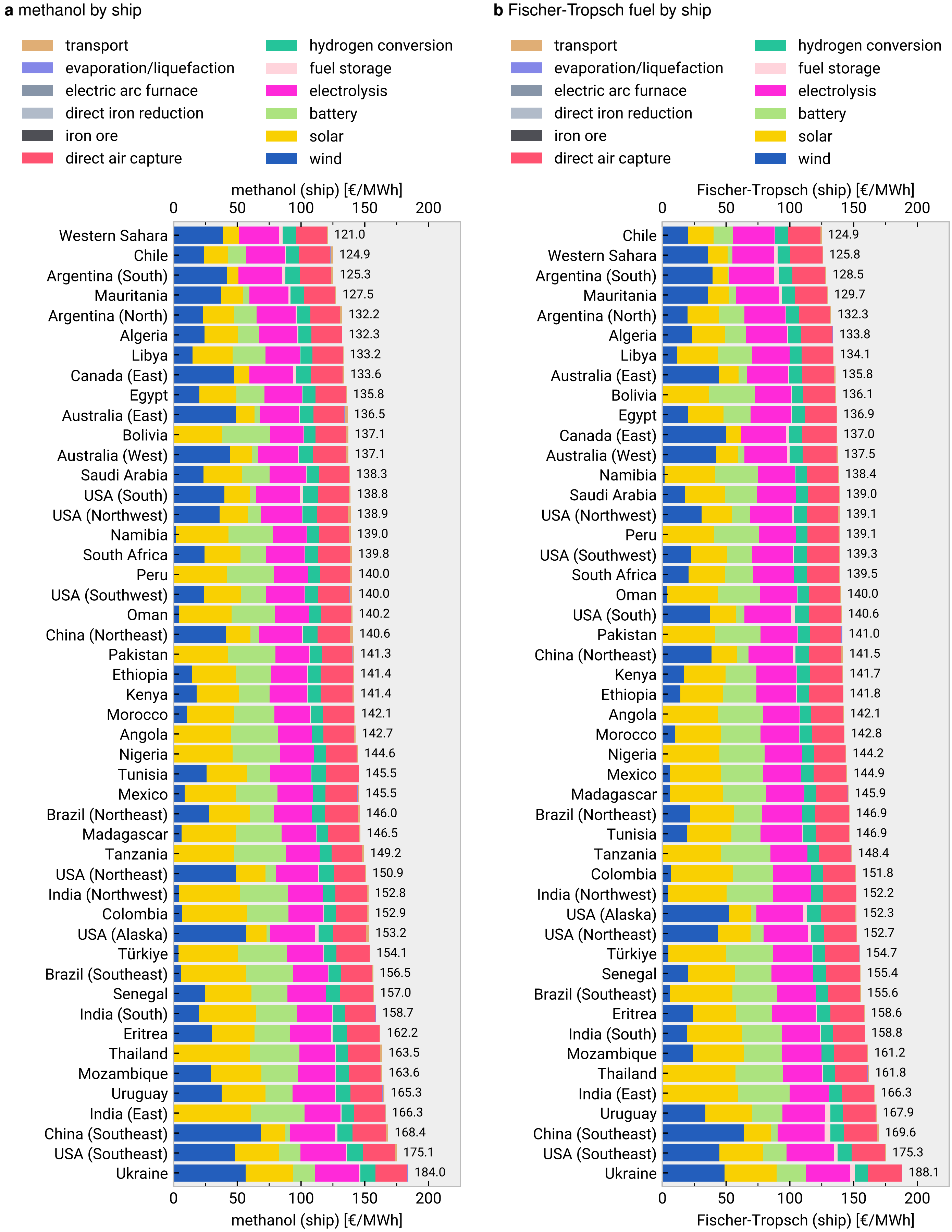


Supplementary Figure 9: **Calculated import cost supply curve for methanol and Fischer-Tropsch by ship.** The levelised cost for each region have been calculated for an annual export volume of 500 TWh. Some landlocked regions in Central Asia have been excluded from ship-based exports.

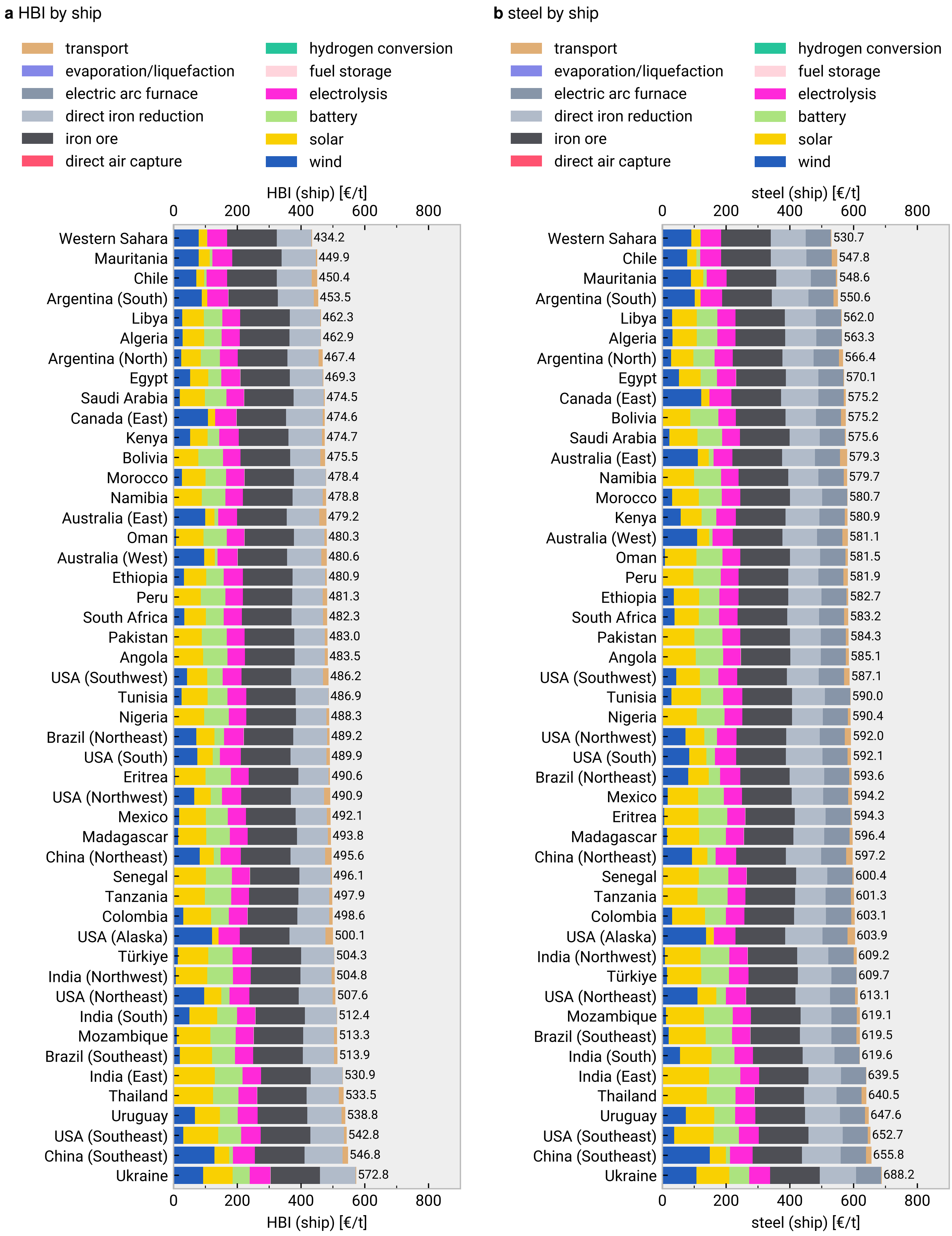


Supplementary Figure 10: **Calculated import cost supply curve for HBI and steel by ship.** The levelised cost for each region have been calculated for an annual export volume of 100 Mt. Some landlocked regions in Central Asia have been excluded from ship-based exports.

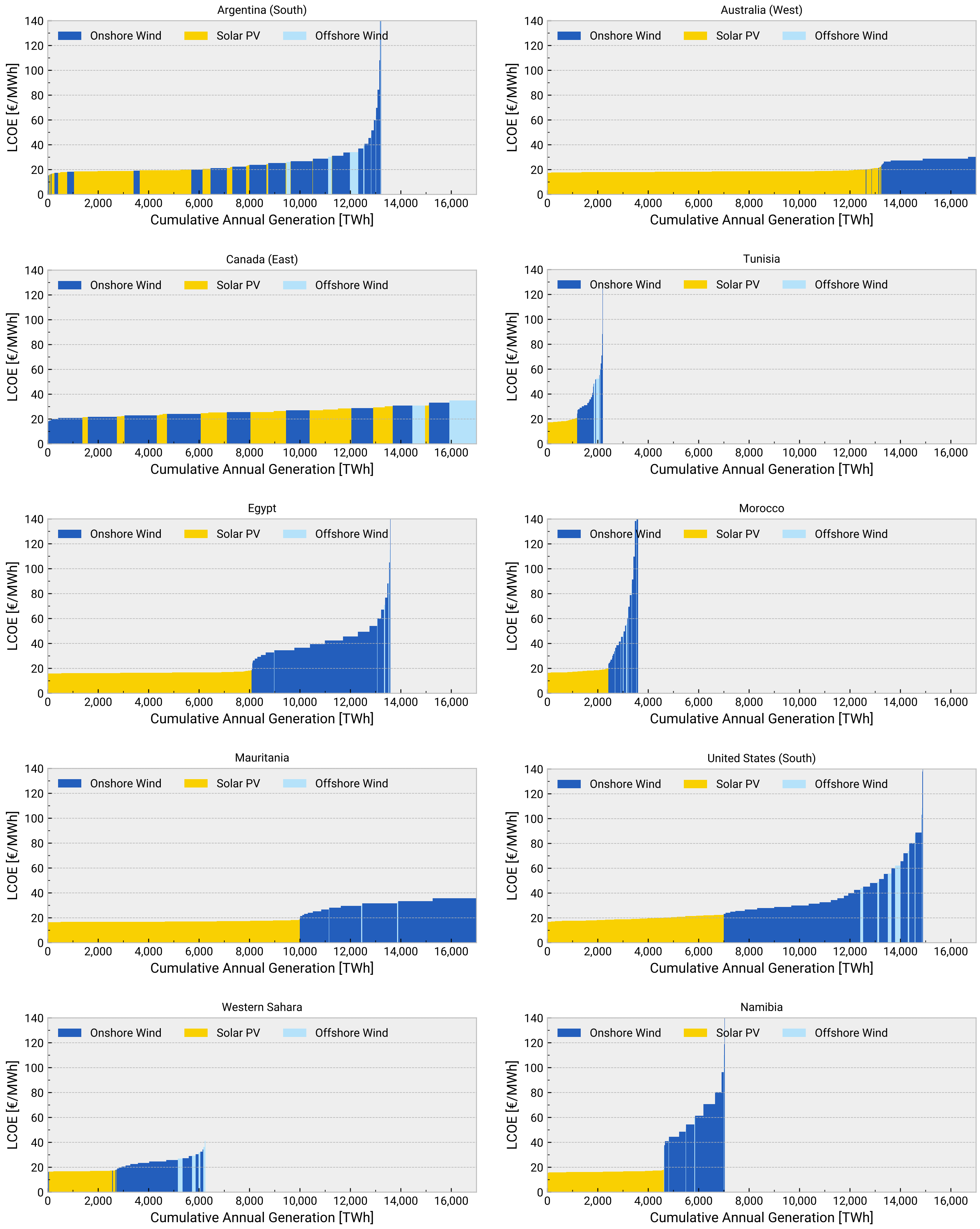


Supplementary Figure 11: **Levelised cost supply curve of electricity in selected exporting regions.** Shows potential and levelised cost of electricity for each resource class of onshore wind (blue), offshore wind (light blue), and solar (yellow) in ascending order.

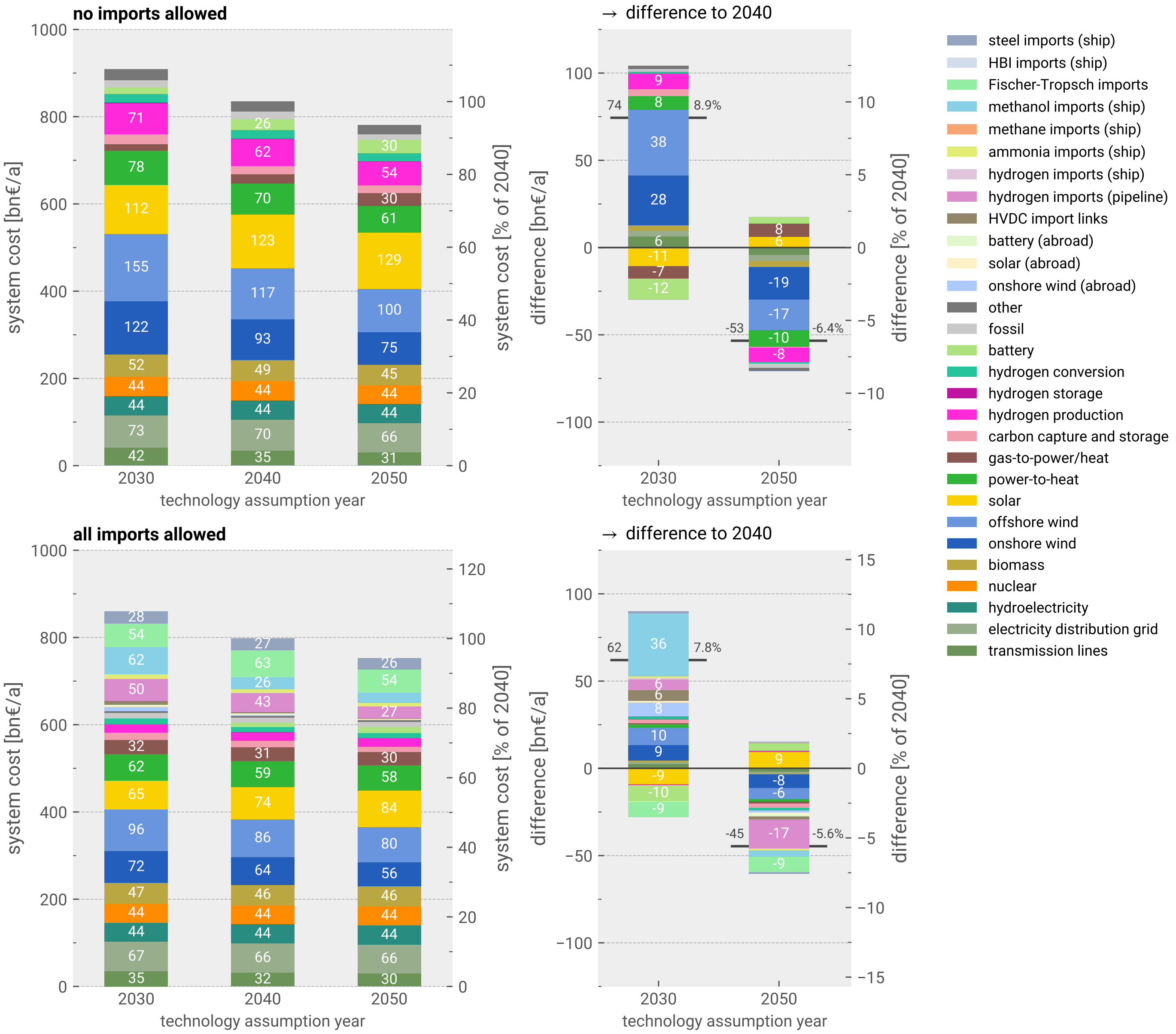


Supplementary Figure 12: **Impact of technology development assumptions on total energy system cost with and without imports.** Left colums show total system cost. Right columns show difference in total system cost compared to technology assumptions for 2040. Black lines show net cost difference in absolute and relative terms. HBI = hot briquetted iron; HVDC = high-voltage direct current.

**a** 10% higher import costs

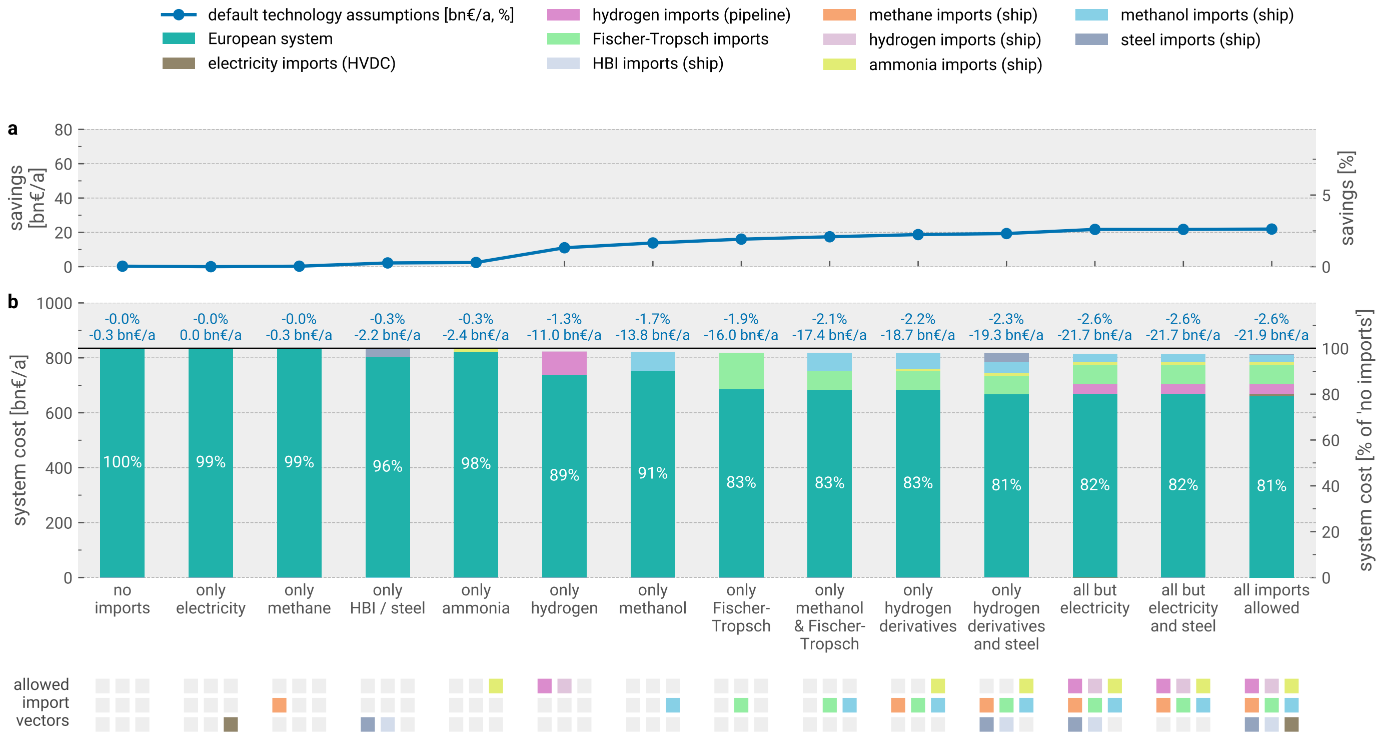


**b** 20% higher import costs

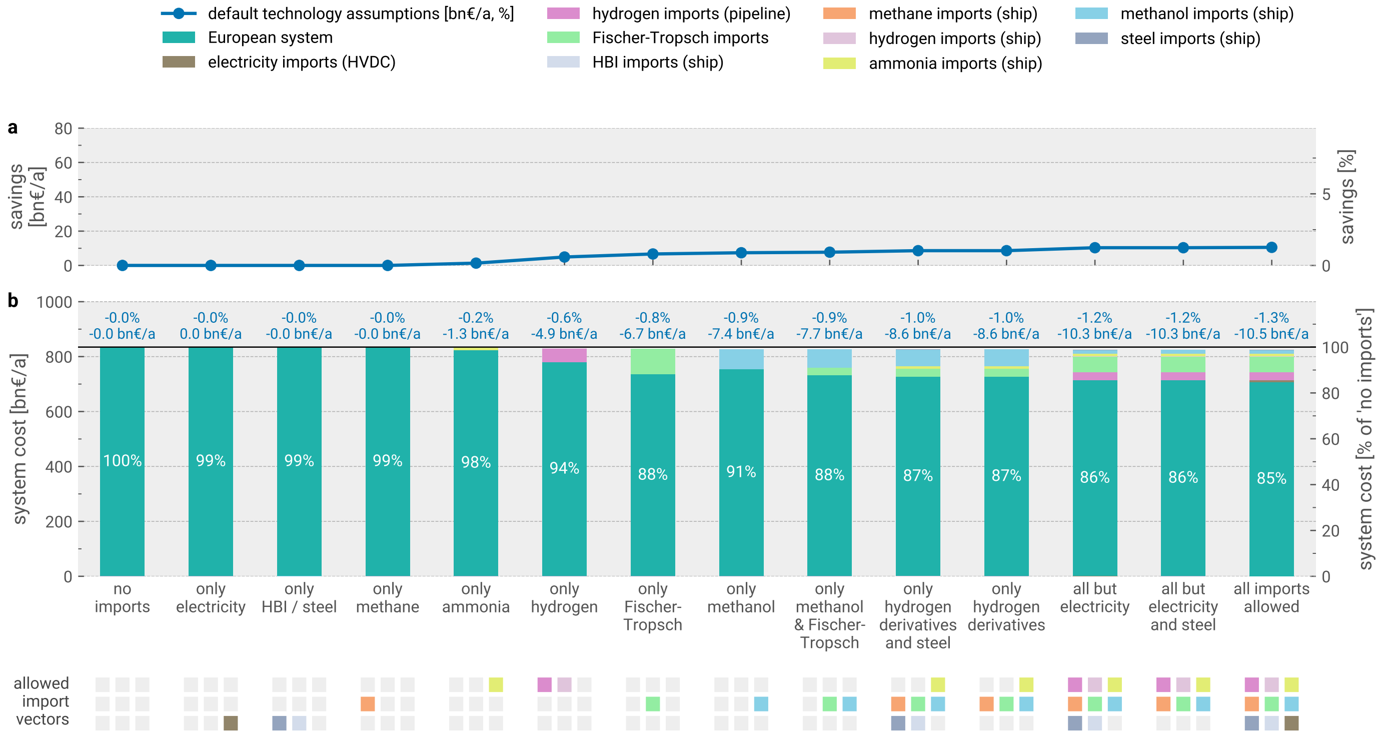


Supplementary Figure 13: **Potential for cost reductions with reduced sets of import options for higher import costs.** This figure includes the sensitivity with **a** 10% and **b** 20% higher import costs for all fuels but electricity. HBI = hot briquetted iron; HVDC = high-voltage direct current.

**a** 10% lower import costs

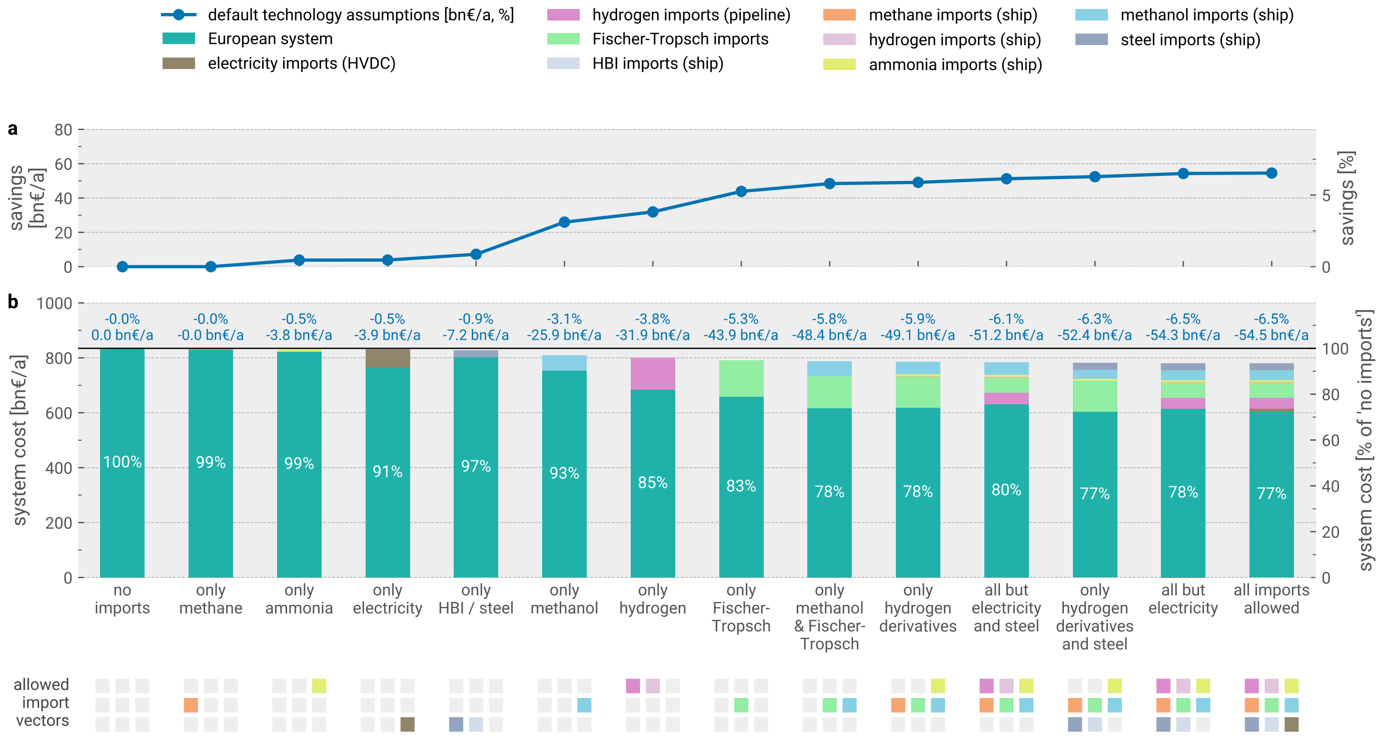


**b** 20% lower import costs

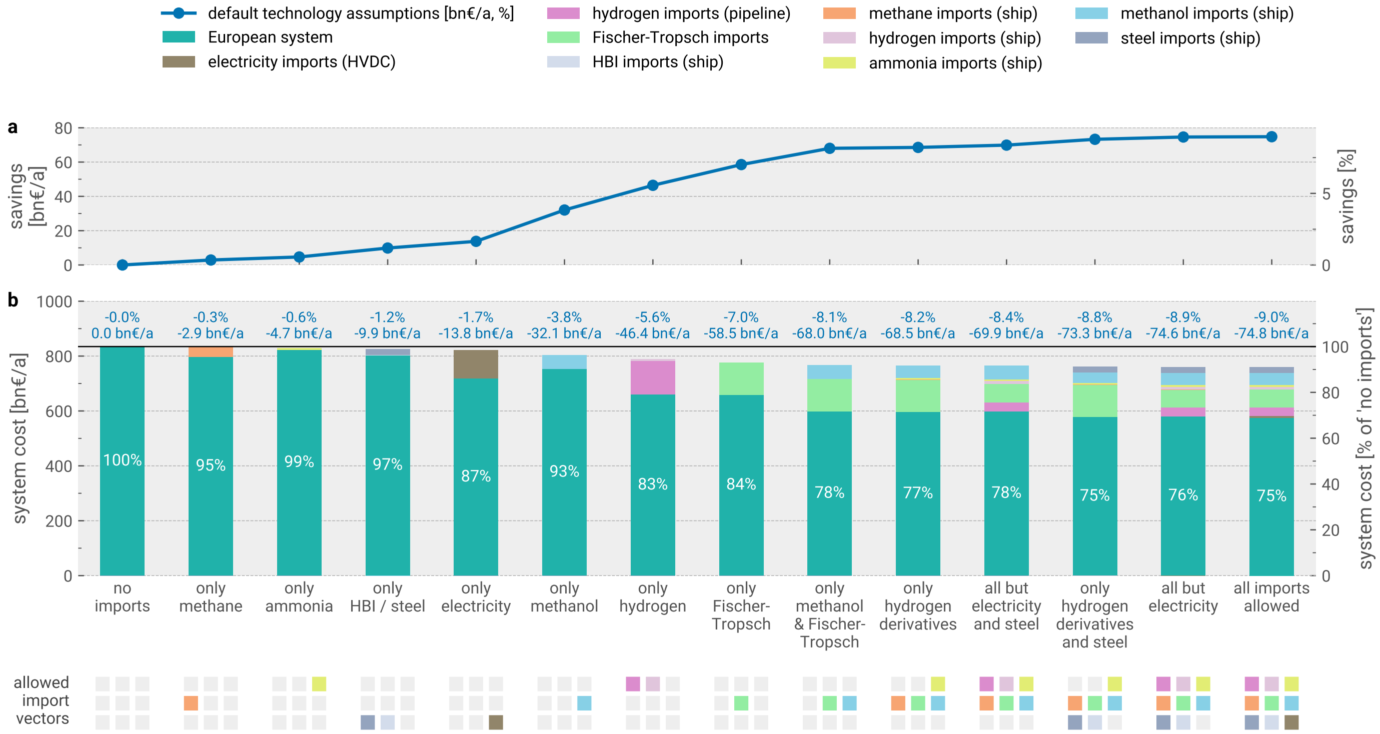


Supplementary Figure 14: **Potential for cost reductions with reduced sets of import options for lower import costs.** This figure includes the sensitivity with **a** 10% and **b** 20% lower import costs for all fuels but electricity. HBI = hot briquetted iron; HVDC = high-voltage direct current.

**a** 50% higher import costs

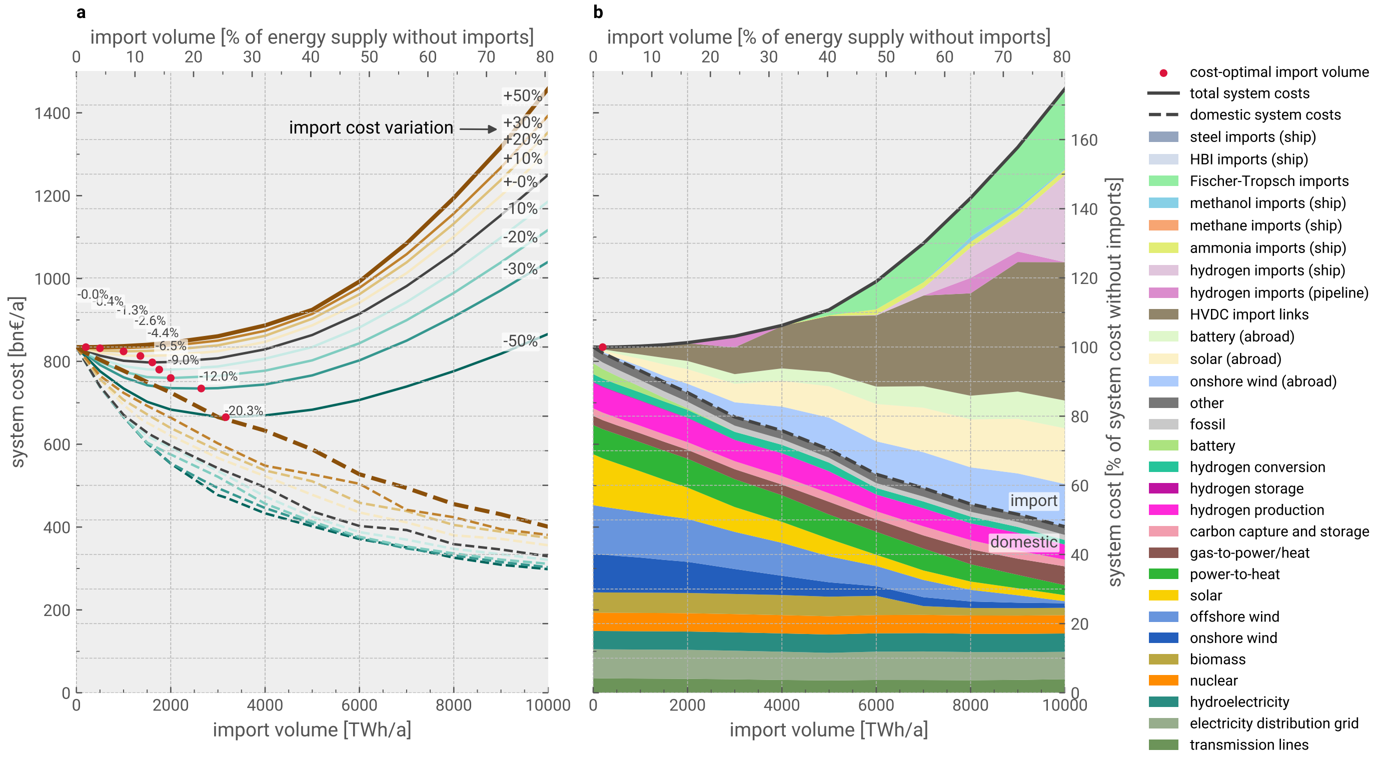


**b** 30% higher import costs

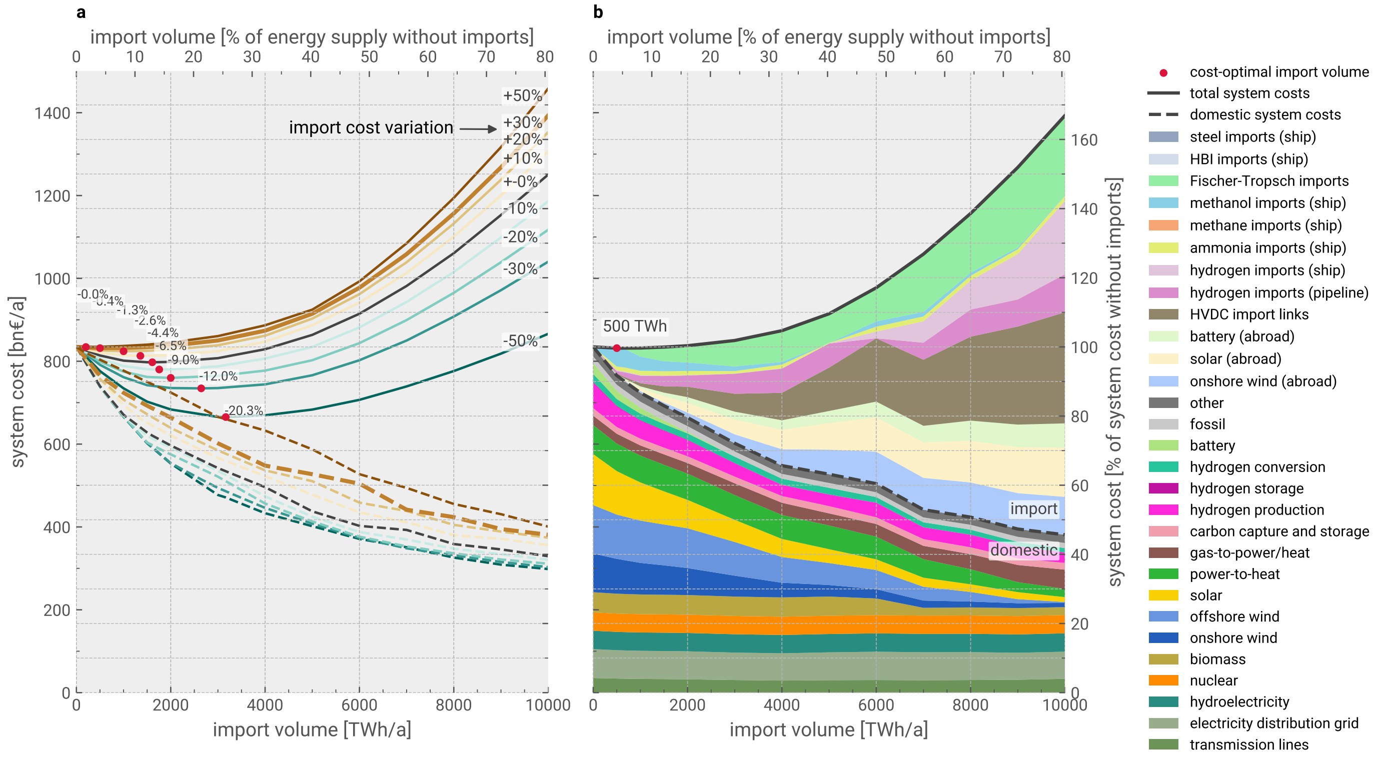


Supplementary Figure 15: **Sensitivity of import volume on total system cost and composition for varying import costs.** This figure includes the sensitivity with **a** 50% and **b** 30% higher import costs for all fuels but electricity. HBI = hot briquetted iron; HVDC = high-voltage direct current.

**a** 20% higher import costs

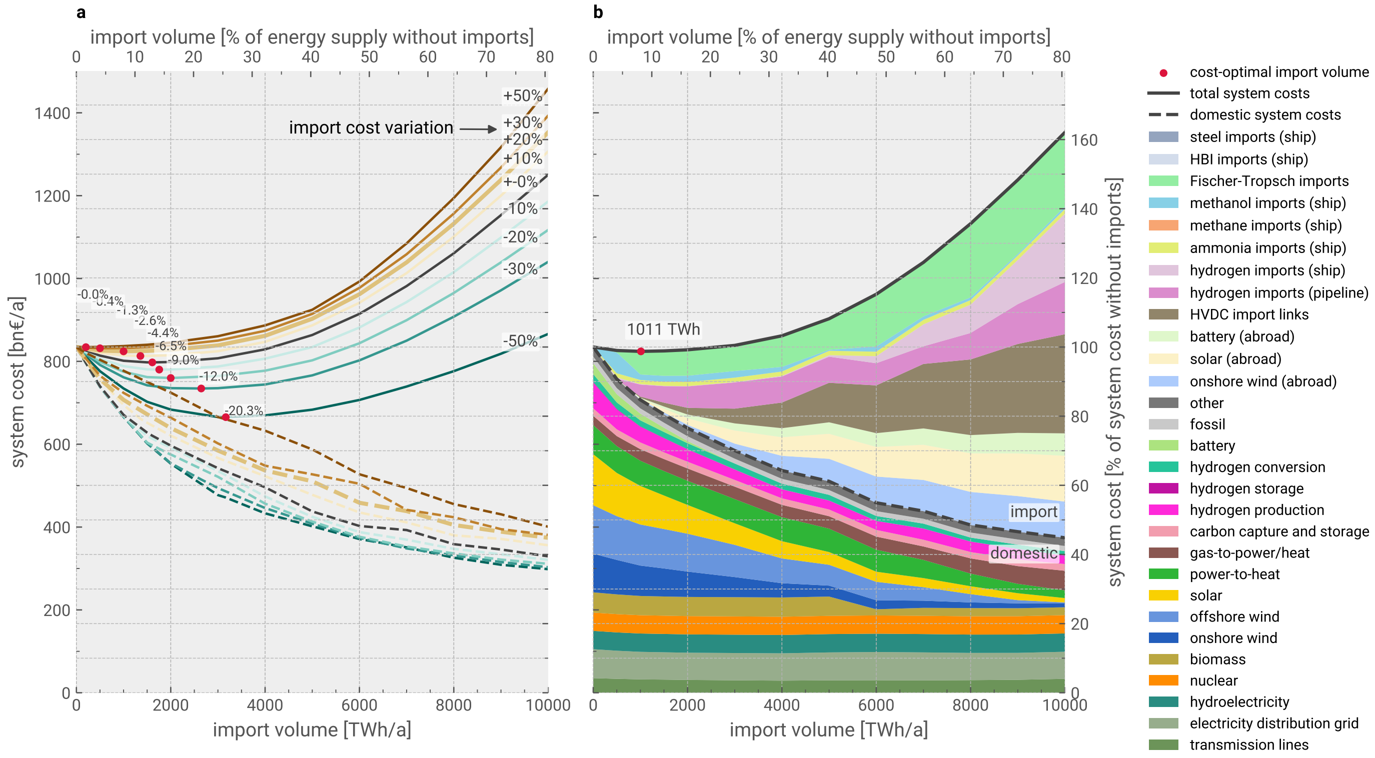


**b** 10% higher import costs

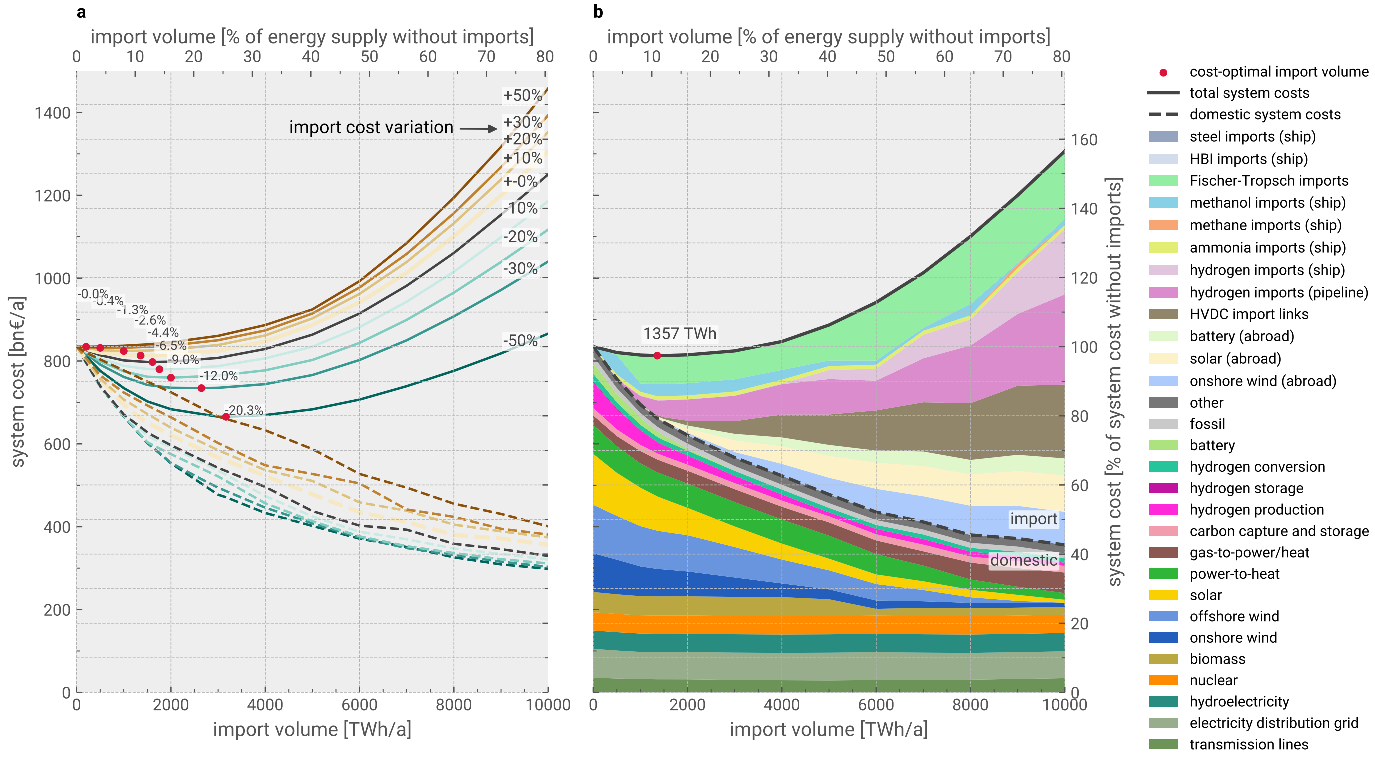


Supplementary Figure 16: **Sensitivity of import volume on total system cost and composition for varying import costs.** This figure includes the sensitivity with **a** 10% and **b** 20% higher import costs for all fuels but electricity. HBI = hot briquetted iron; HVDC = high-voltage direct current.

**a** 10% lower import costs

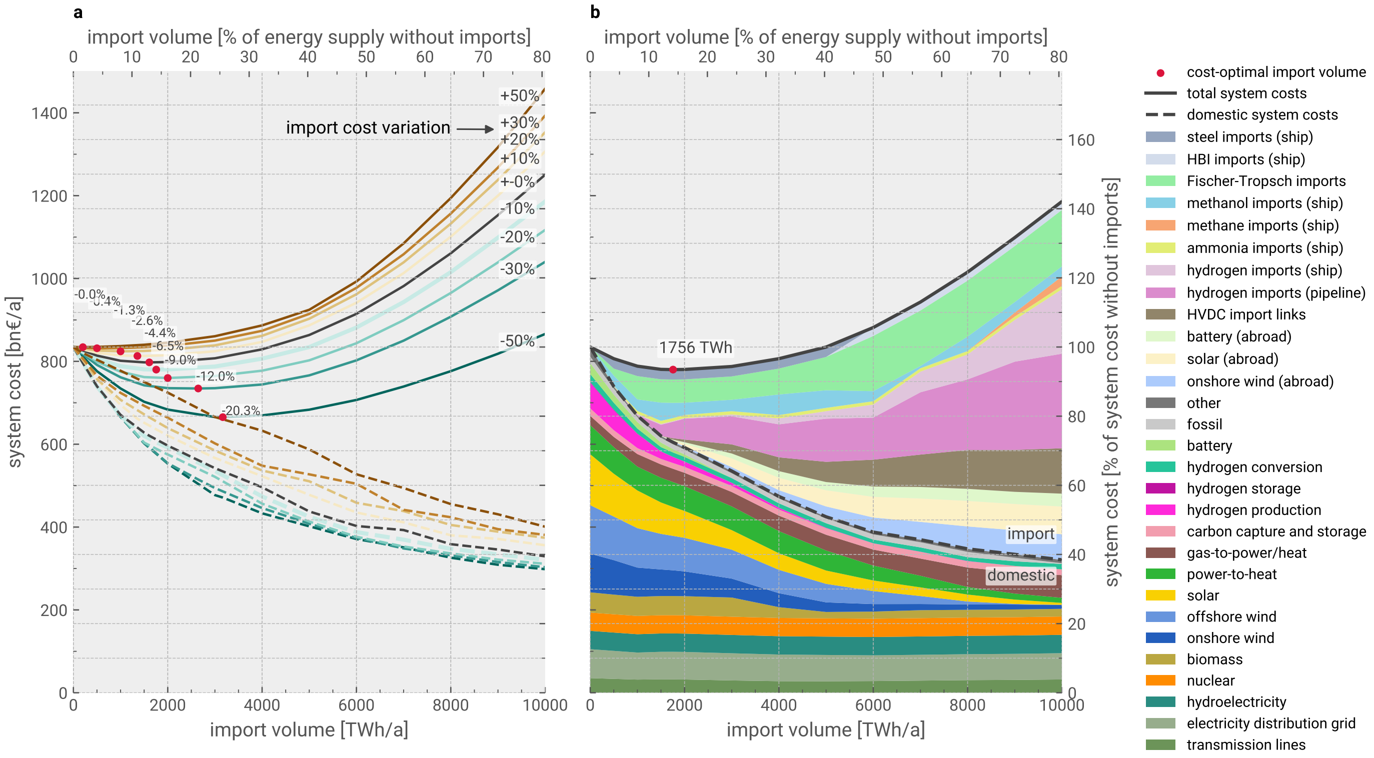


**b** 20% lower import costs

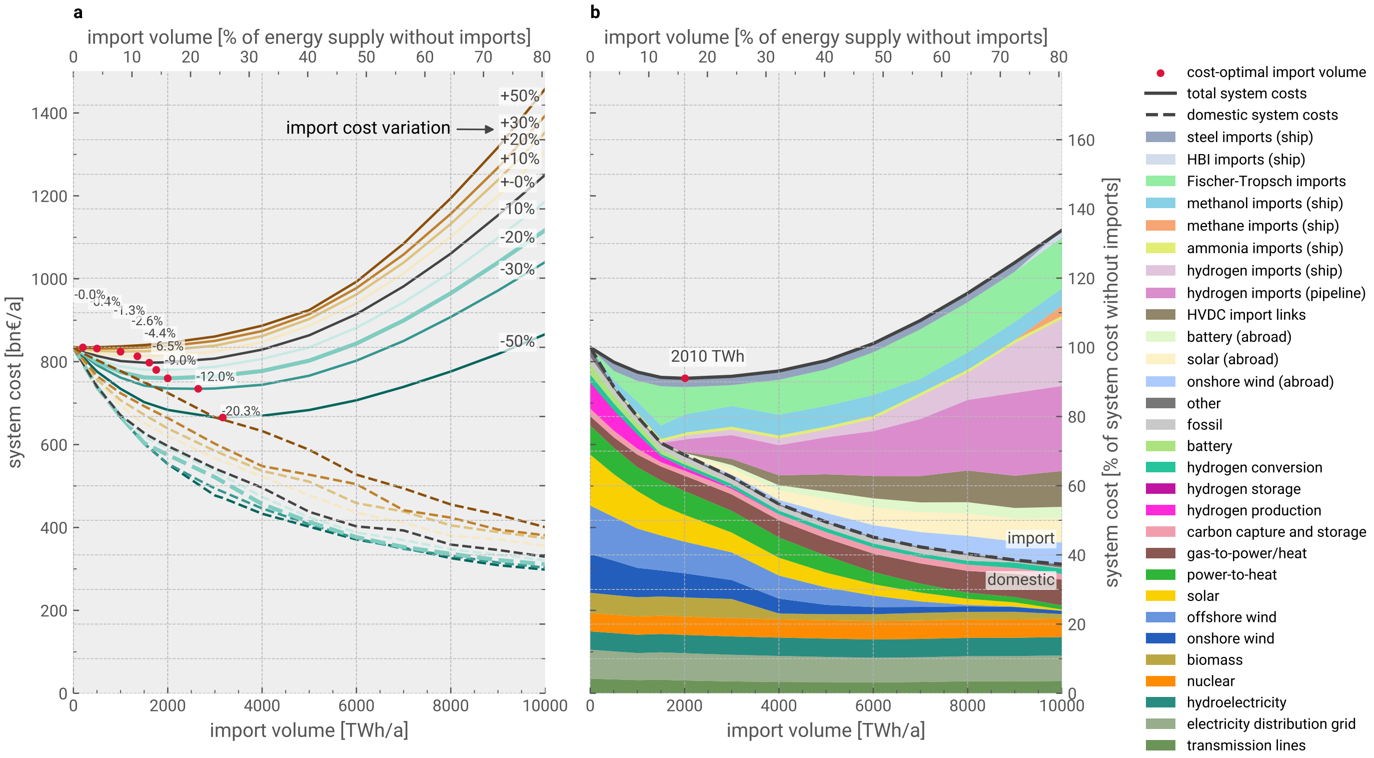


Supplementary Figure 17: **Sensitivity of import volume on total system cost and composition for varying import costs.** This figure includes the sensitivity with **a** 10% and **b** 20% lower import costs for all fuels but electricity. HBI = hot briquetted iron; HVDC = high-voltage direct current.

**a** 30% lower import costs

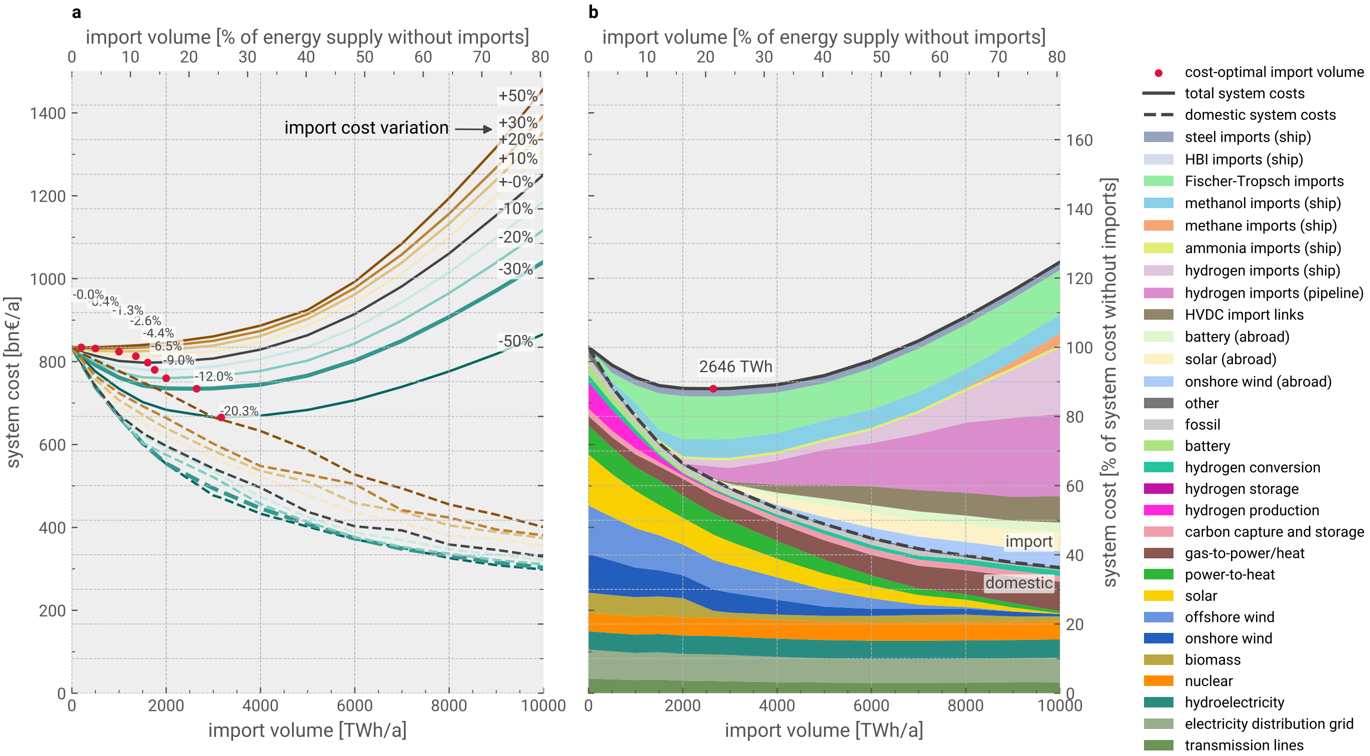


**b** 50% lower import costs

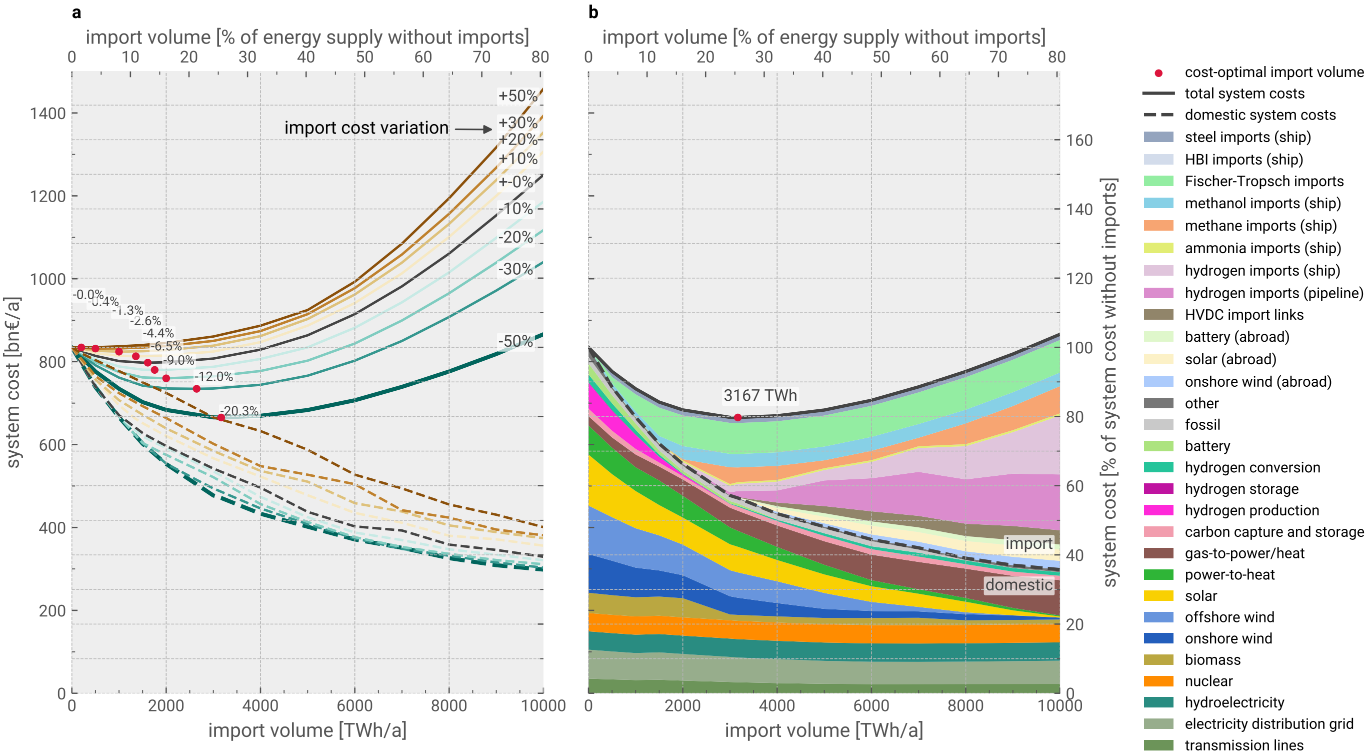


Supplementary Figure 18: **Sensitivity of import volume on total system cost and composition for varying import costs.** This figure includes the sensitivity with **a** 30% and **b** 50% lower import costs for all fuels but electricity. HBI = hot briquetted iron; HVDC = high-voltage direct current.

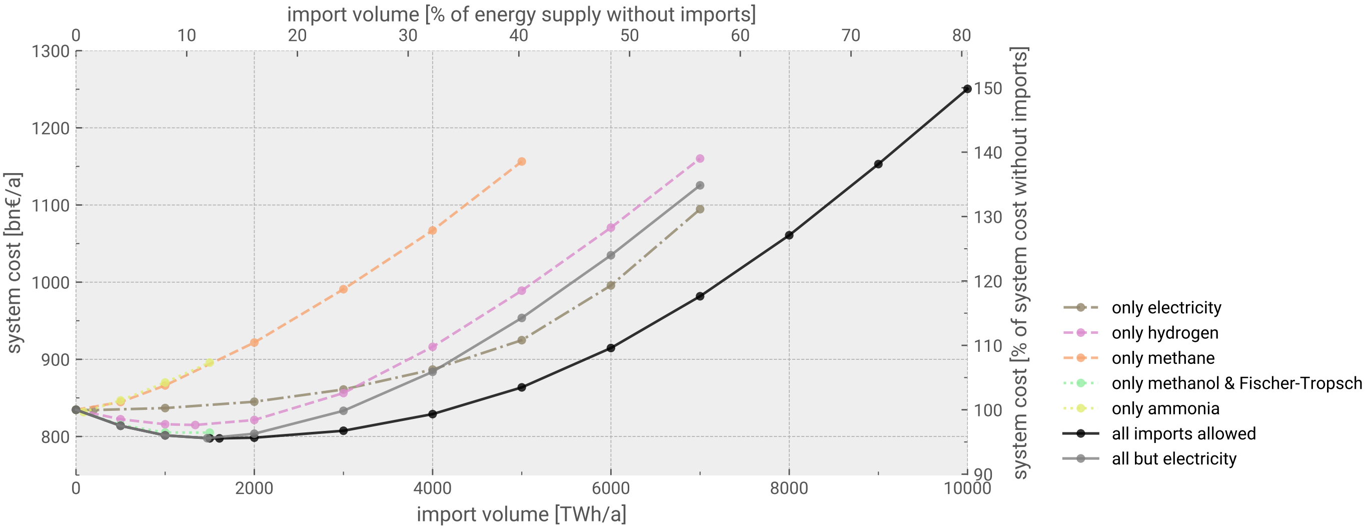


Supplementary Figure 19: **Sensitivity of import volume on total system cost with subsets of import vectors available.** Supplement to Figure 6. The volume of imports is exogenously forced for these runs and coloured lines represent certain restrictions in available import vectors.

**a** only electricity imports

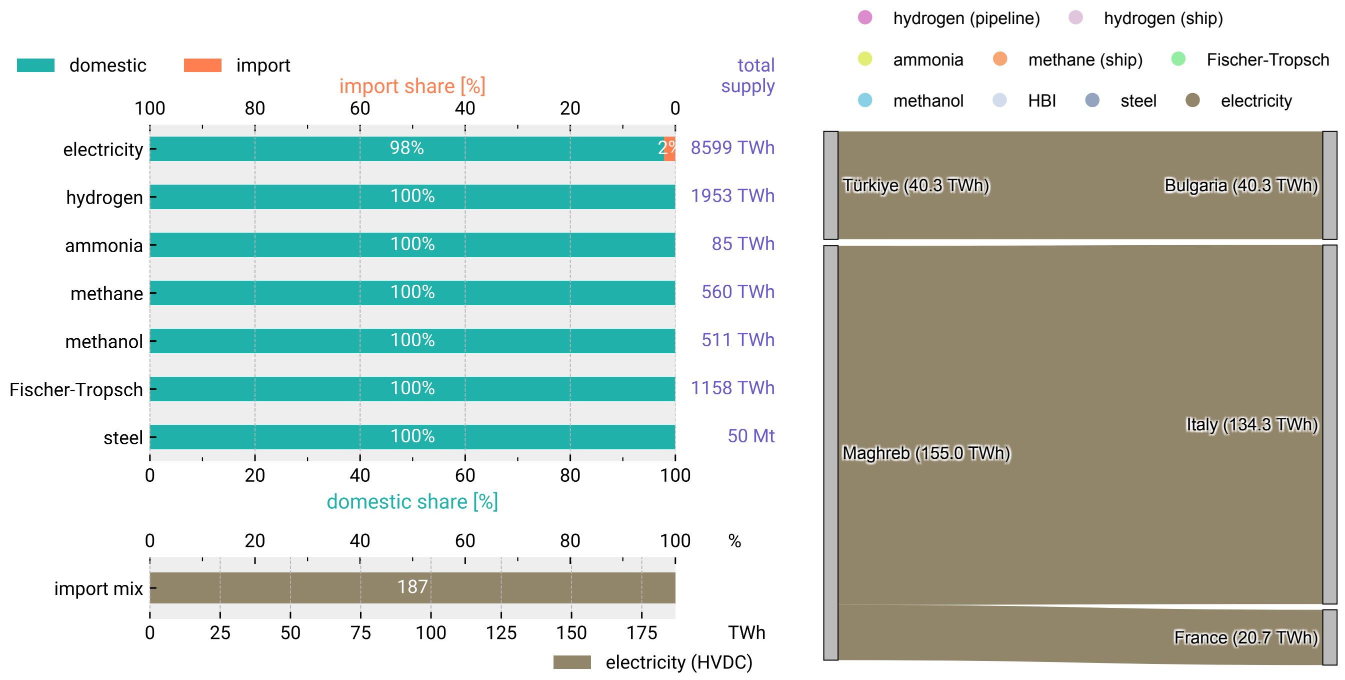


**b** only hydrogen imports

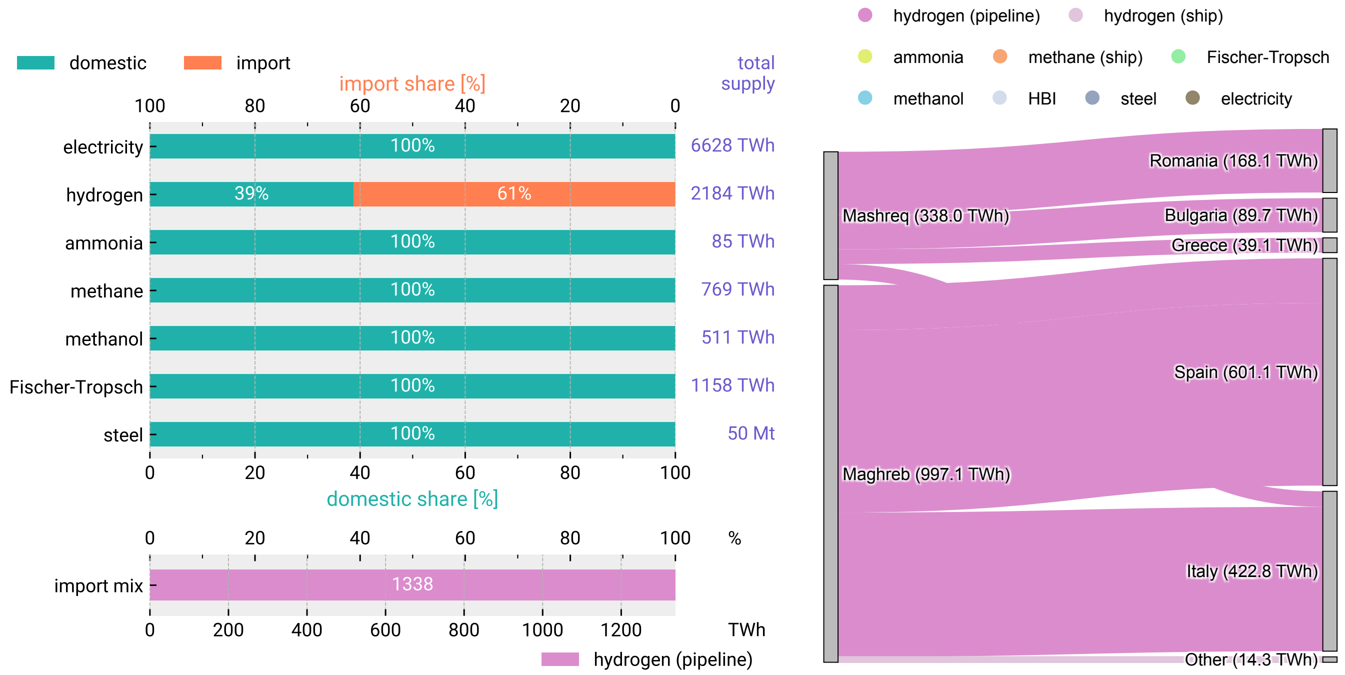


Supplementary Figure 20: **Import shares, mix and trade flows for import scenarios with restricted import vectors.** For **a** only electricity imports and **b** only hydrogen imports. Supplement to Figure 3. HBI = hot briquetted iron.

**a** 10% lower import costs (all carriers but electricity)

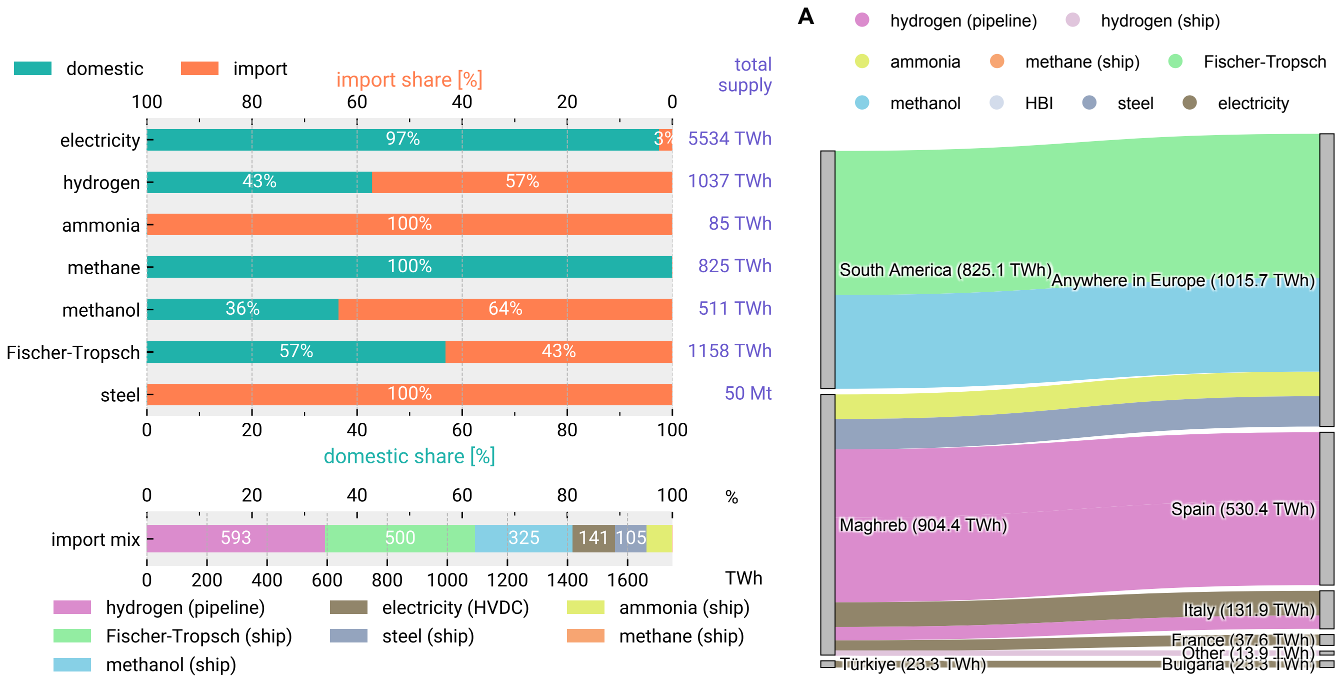


**b** 10% lower import cost (only carbonaceous fuels)

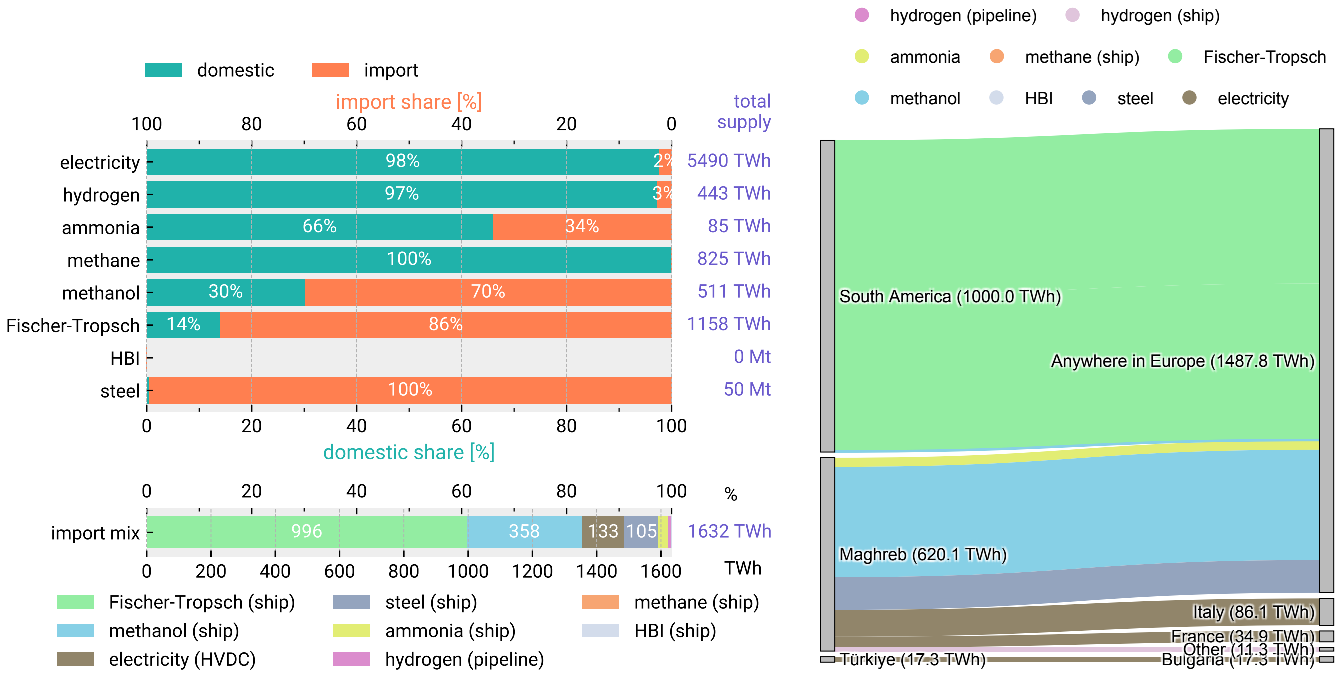


Supplementary Figure 21: **Import shares, mix and trade flows for import scenarios with 10% lower costs.** For **a** all carries but electricity and **b** only carbonaceous fuels. Supplement to Figure 3. HBI = hot briquetted iron; HVDC = high-voltage direct current.

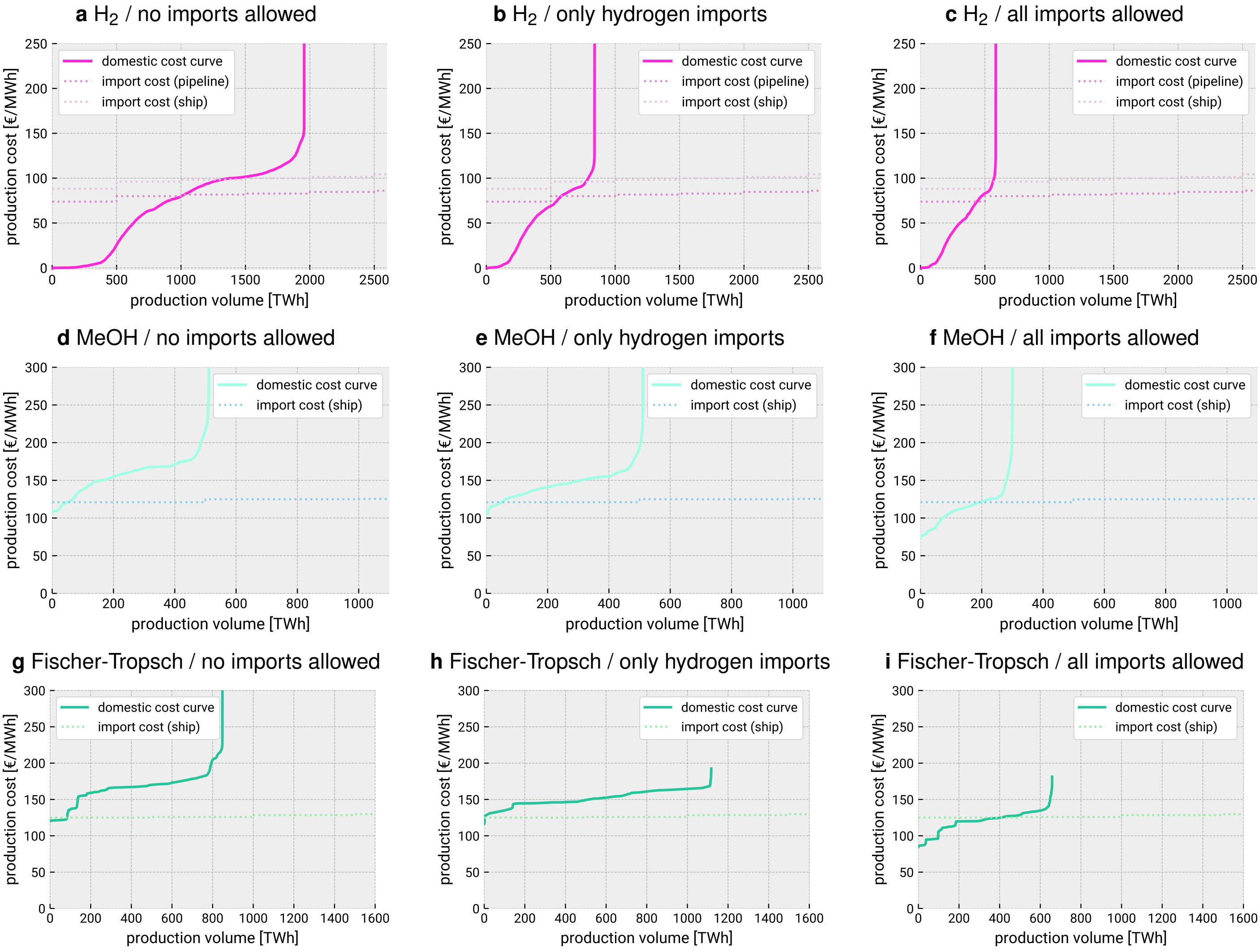


Supplementary Figure 22: **Domestic cost supply curves for different import scenarios and carriers.** The cost supply curves for hydrogen (**a,b,c**), methanol (**d,e,f**), and Fischer-Tropsch (**g,h,i**) are built using sorted spatio-temporal market values with corresponding production volumes per region and snapshot for scenarios without imports (**a,d,g**), only hydrogen imports (**b,e,h**), and all imports allowed (**c,f,i**). Dotted lines show the import cost supply curves (in steps of 500 TWh) of the respective carriers as reference.

**a** no imports allowed

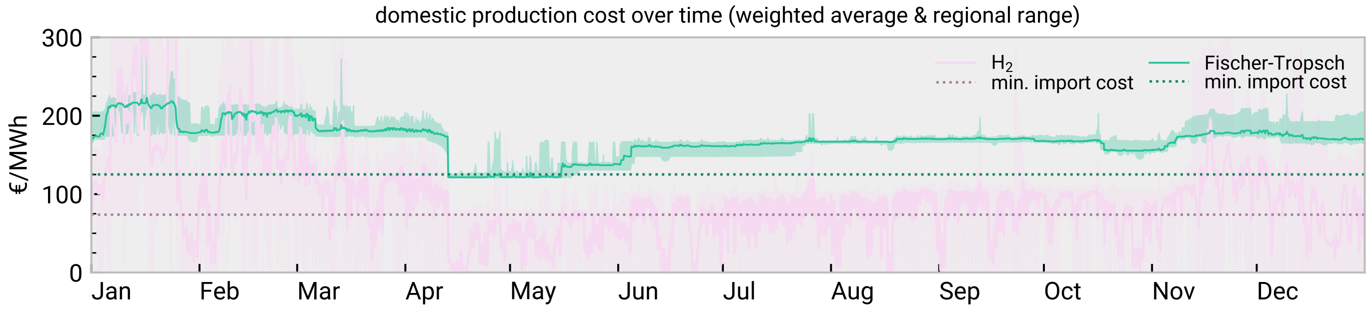


**b** only hydrogen imports allowed

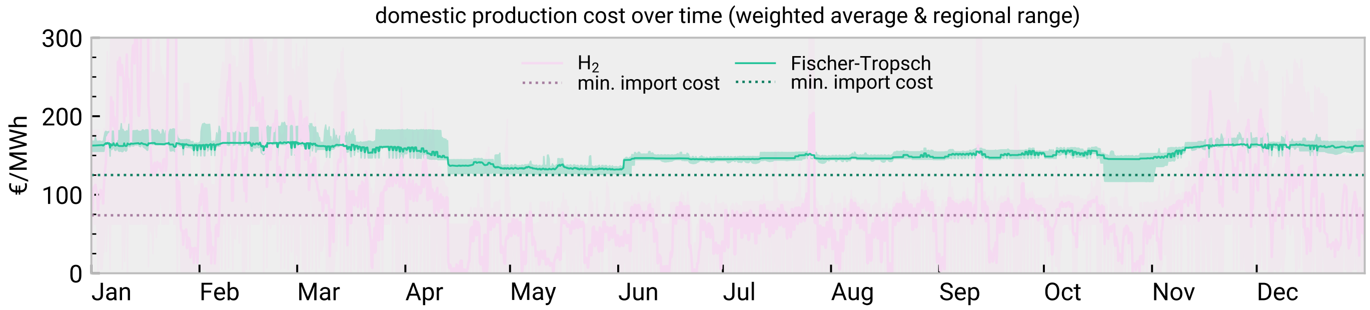


**c** all imports allowed

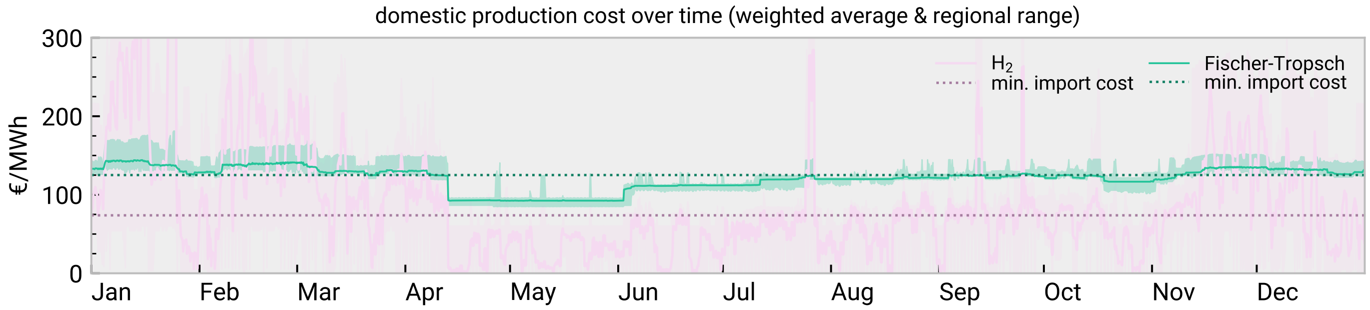


Supplementary Figure 23: **Temporal variations of domestic hydrogen and Fischer-Tropsch production costs for different import scenarios.** Supplement to Figure 4. Scenarios shown include no imports (**a**), only hydrogen imports (**b**), and all imports allowed (**c**). Dotted lines show the minimum import costs as a reference. The solid lines show the production-weighted average costs across all regions. The shaded areas show the regional range between lowest and highest production cost in any of the 115 model regions.

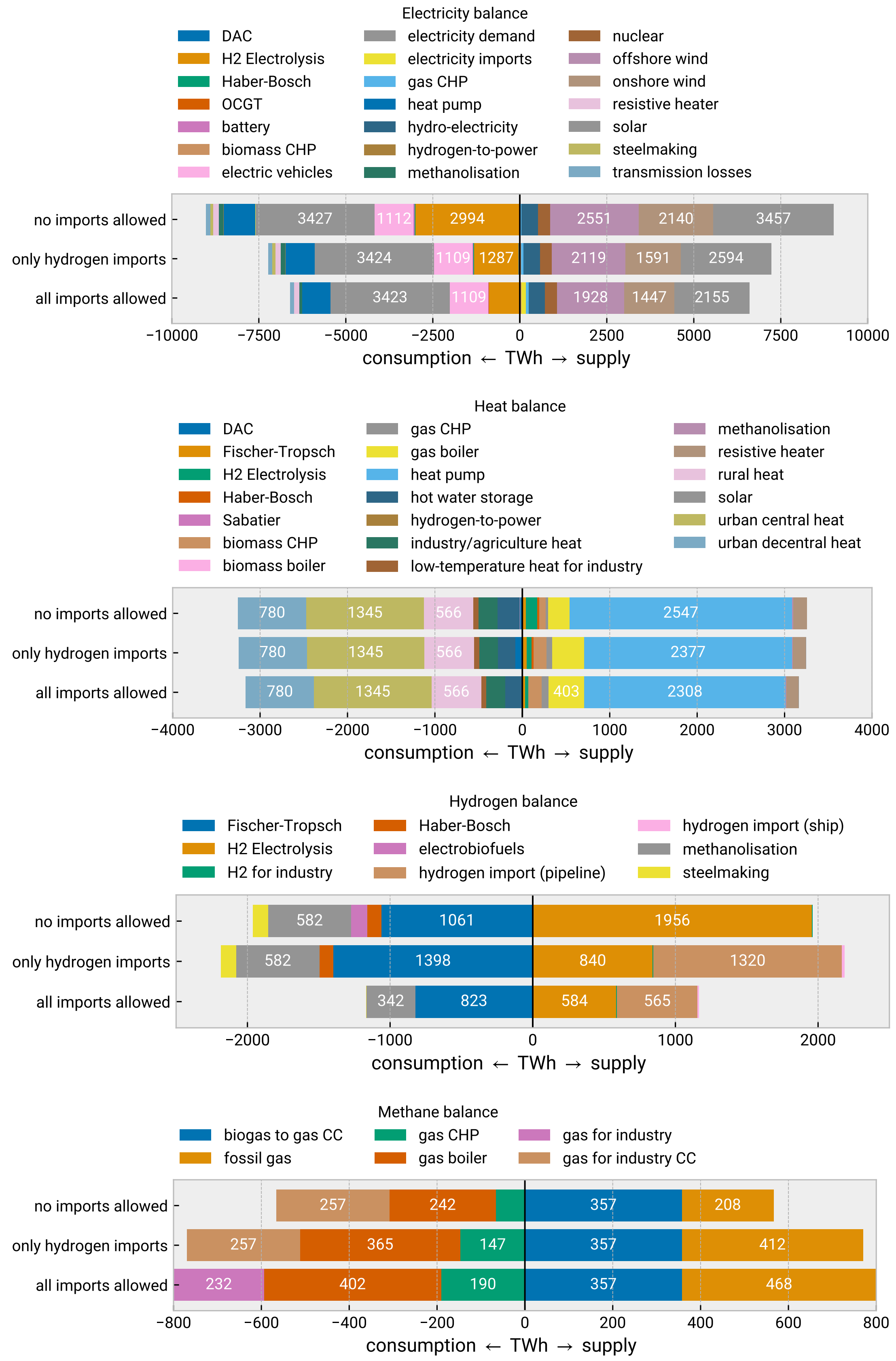


Supplementary Figure 24: **Energy balances for three import scenarios for the carriers electricity, heat, hydrogen and gas.** DAC = direct air capture; OCGT = open cycle gas turbine; CHP = combined heat and power; CC = carbon capture.

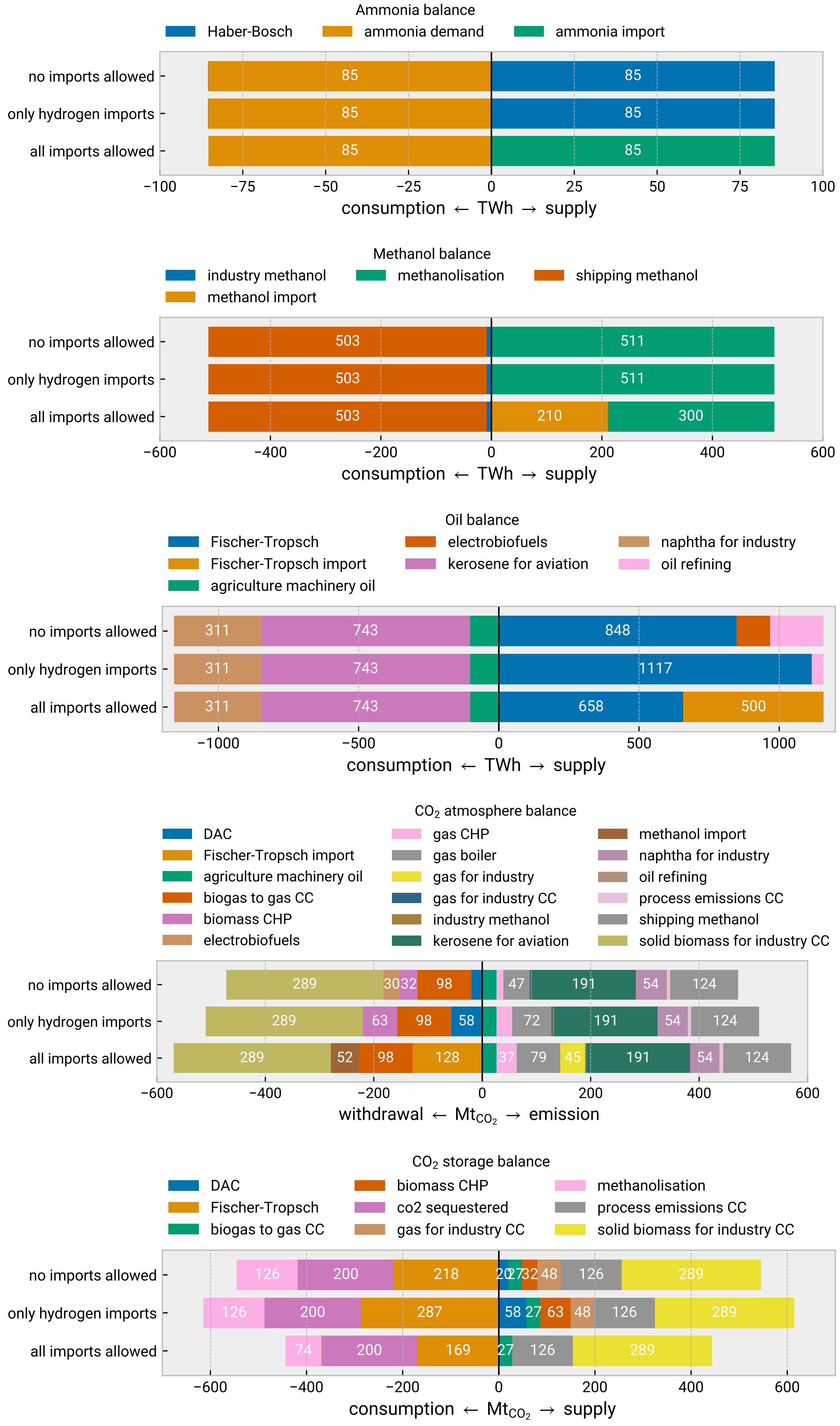


Supplementary Figure 25: **Energy balances for three import scenarios for the carriers ammonia, methanol, and oil, as well as stored and atmospheric carbon dioxide.** CC = carbon capture; DAC = direct air capture; CHP = combined heat and power.

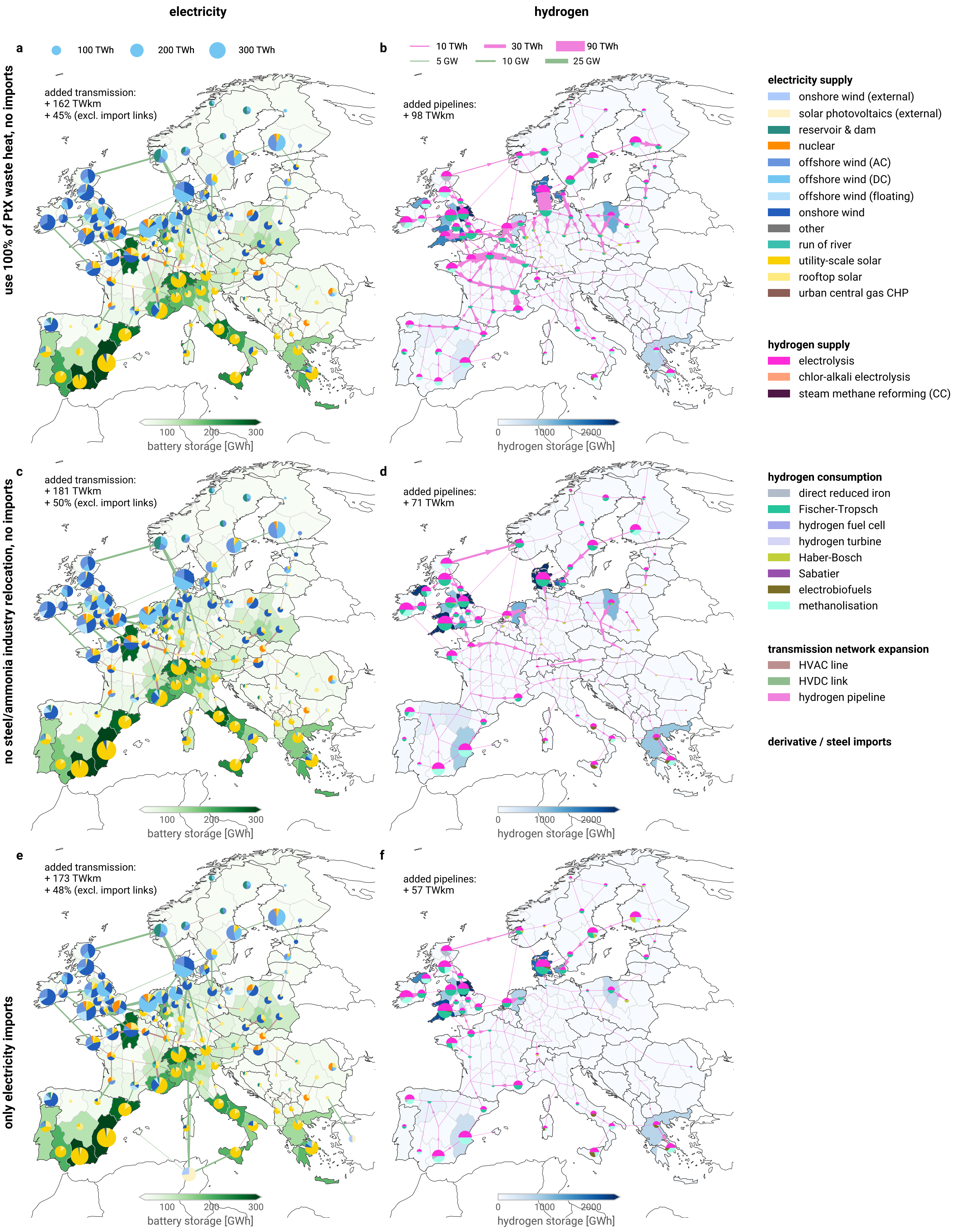


Supplementary Figure 26: **Layout of European energy infrastructure for different import scenarios.** Infrastructure build-out with full power-to-X waste heat availability in domestic scenario (**a**,**b**), without steel and ammonia industry relocation (**c**,**d**), and only electricity imports (**e**,**f**). **a**,**c**,**e** show the regional electricity supply mix (pies), added HVDC and HVAC transmission capacity (lines), and the siting of battery storage (choropleth). **b**,**d**,**f** show the hydrogen supply (top half of pies) and consumption (bottom half of pies), net flow and direction of hydrogen in newly built and retrofitted pipelines (lines), and the siting of hydrogen storage subject to geological potentials (choropleth). Total volumes of transmission expansion are given in TWkm, which is the sum product of the capacity and length of individual connections. Maps made with Natural Earth. HBI = hot briquetted iron; HVDC = high-voltage direct current; HVAC = high-voltage alternating current; AC = alternating current; DC = direct current; CHP = combined heat and power.

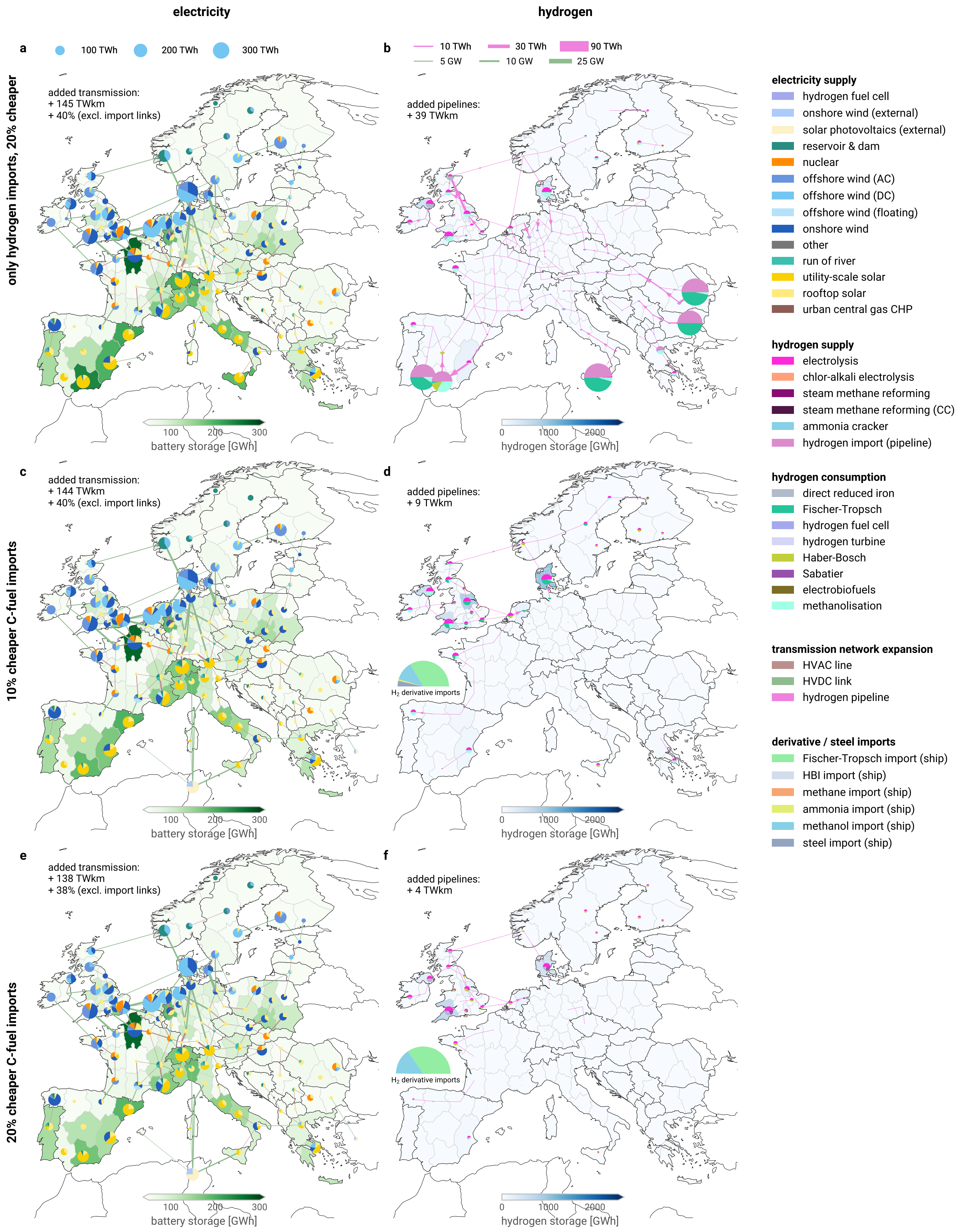


Supplementary Figure 27: **Layout of European energy infrastructure for different import scenarios.** Sensitivities of infrastructure build-out to import costs. **a,c,e** show the regional electricity supply mix (pies), added HVDC and HVAC transmission capacity (lines), and the siting of battery storage (choropleth). **b,d,f** show the hydrogen supply (top half of pies) and consumption (bottom half of pies), net flow and direction of hydrogen in newly built and retrofitted pipelines (lines), and the siting of hydrogen storage subject to geological potentials (choropleth). Total volumes of transmission expansion are given in TWkm, which is the sum product of the capacity and length of individual connections. Maps made with Natural Earth. HBI = hot briquetted iron; HVDC = high-voltage direct current; HVAC = high-voltage alternating current; AC = alternating current; DC = direct current; CHP = combined heat and power.

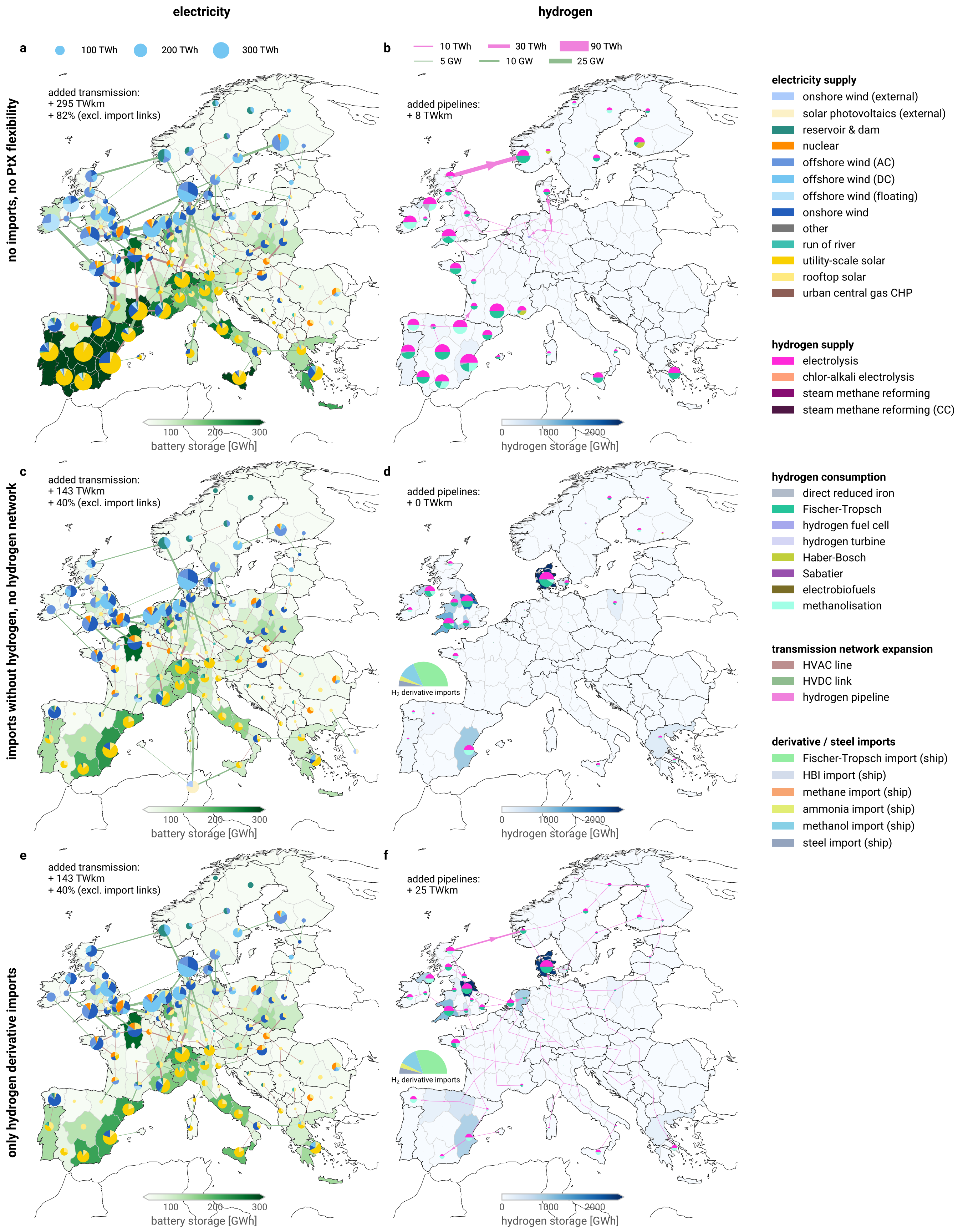


Supplementary Figure 28: **Layout of European energy infrastructure for different import scenarios.** Infrastructure build-out with no power-to-X flexibility (**a**,**b**), without hydrogen imports or pipeline network (**c**,**d**), only hydrogen derivative imports (**e**,**f**). **a**,**c**,**e** show the regional electricity supply mix (pies), added HVDC and HVAC transmission capacity (lines), and the siting of battery storage (choropleth). **b**,**d**,**f** show the hydrogen supply (top half of pies) and consumption (bottom half of pies), net flow and direction of hydrogen in newly built and retrofitted pipelines (lines), and the siting of hydrogen storage subject to geological potentials (choropleth). Total volumes of transmission expansion are given in TWkm, which is the sum product of the capacity and length of individual connections. Maps made with Natural Earth. HBI = hot briquetted iron; HVDC = high-voltage direct current; HVAC = high-voltage alternating current; AC = alternating current; DC = direct current; CHP = combined heat and power.

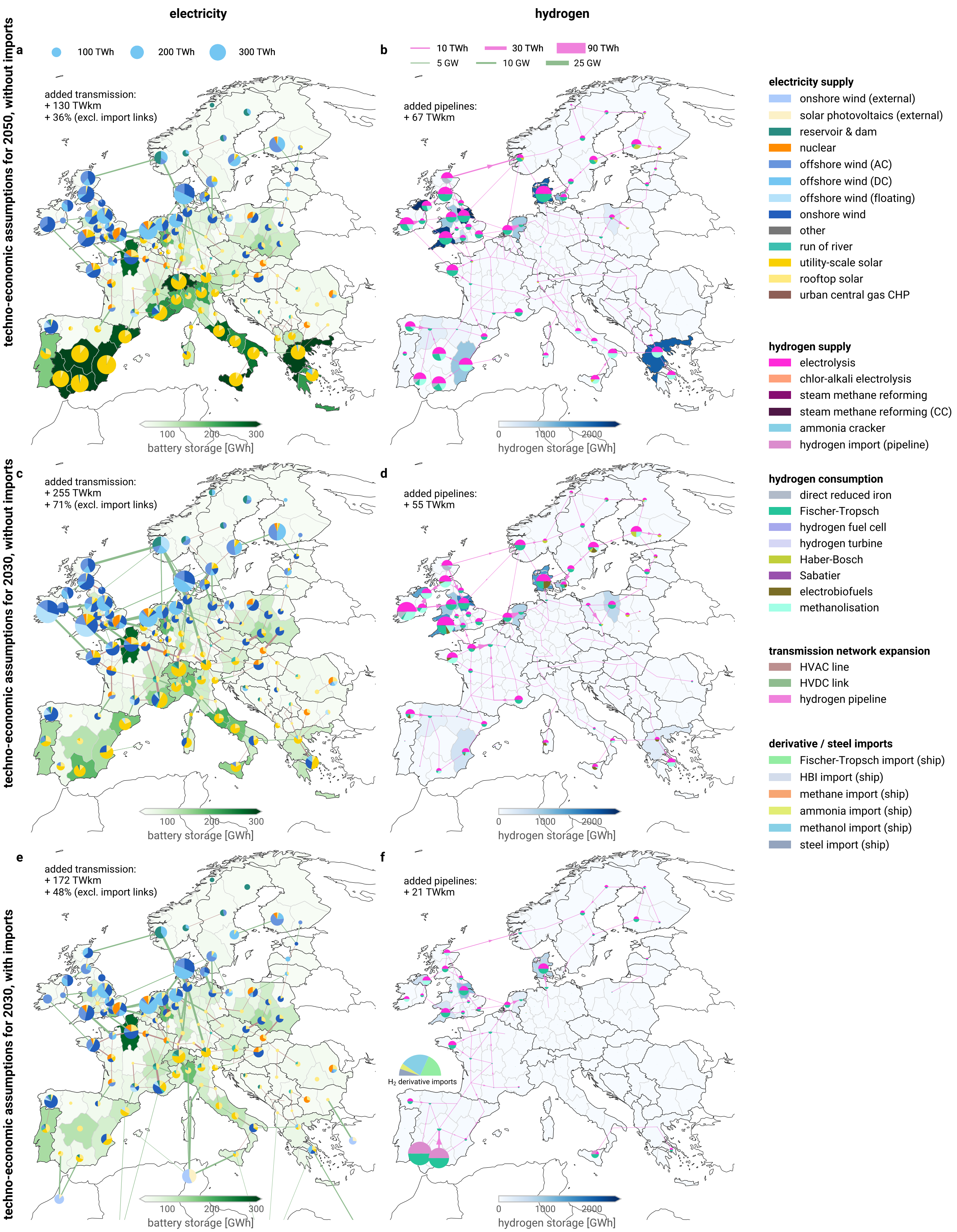


Supplementary Figure 29: **Layout of European energy infrastructure for different import scenarios.** Infrastructure build-out with technology assumptions for 2050 and no imports (**a**,**b**), with technology assumptions for 2030 and no imports (**c**,**d**), with technology assumptions for 2050 and all imports allowed (**e**,**f**). **a**,**c**,**e** show the regional electricity supply mix (pies), added HVDC and HVAC transmission capacity (lines), and the siting of battery storage (choropleth). **b**,**d**,**f** show the hydrogen supply (top half of pies) and consumption (bottom half of pies), net flow and direction of hydrogen in newly built and retrofitted pipelines (lines), and the siting of hydrogen storage subject to geological potentials (choropleth). Total volumes of transmission expansion are given in TWkm, which is the sum product of the capacity and length of individual connections. Maps made with Natural Earth. HBI = hot briquetted iron; HVDC = high-voltage direct current; HVAC = high-voltage alternating current; AC = alternating current; DC = direct current; CHP = combined heat and power.

**a** average utilisation rate of import HVDC links

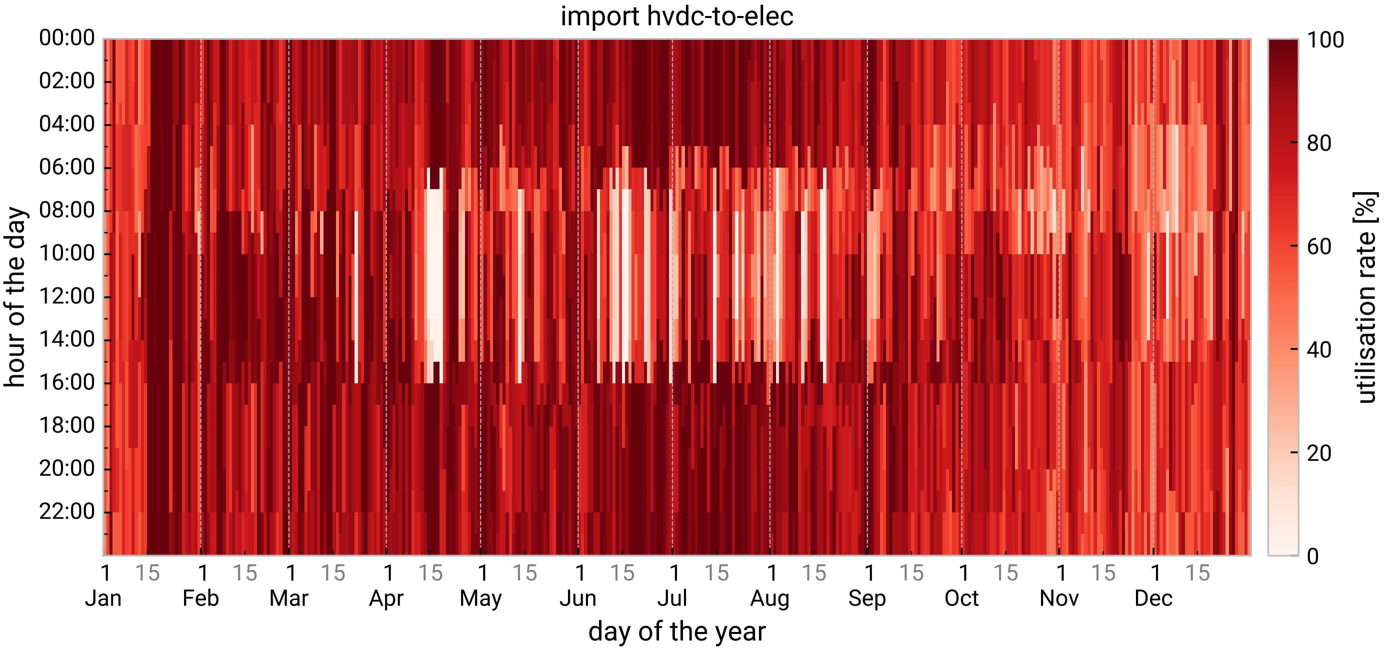


**b** average utilisation rate of import hydrogen pipelines

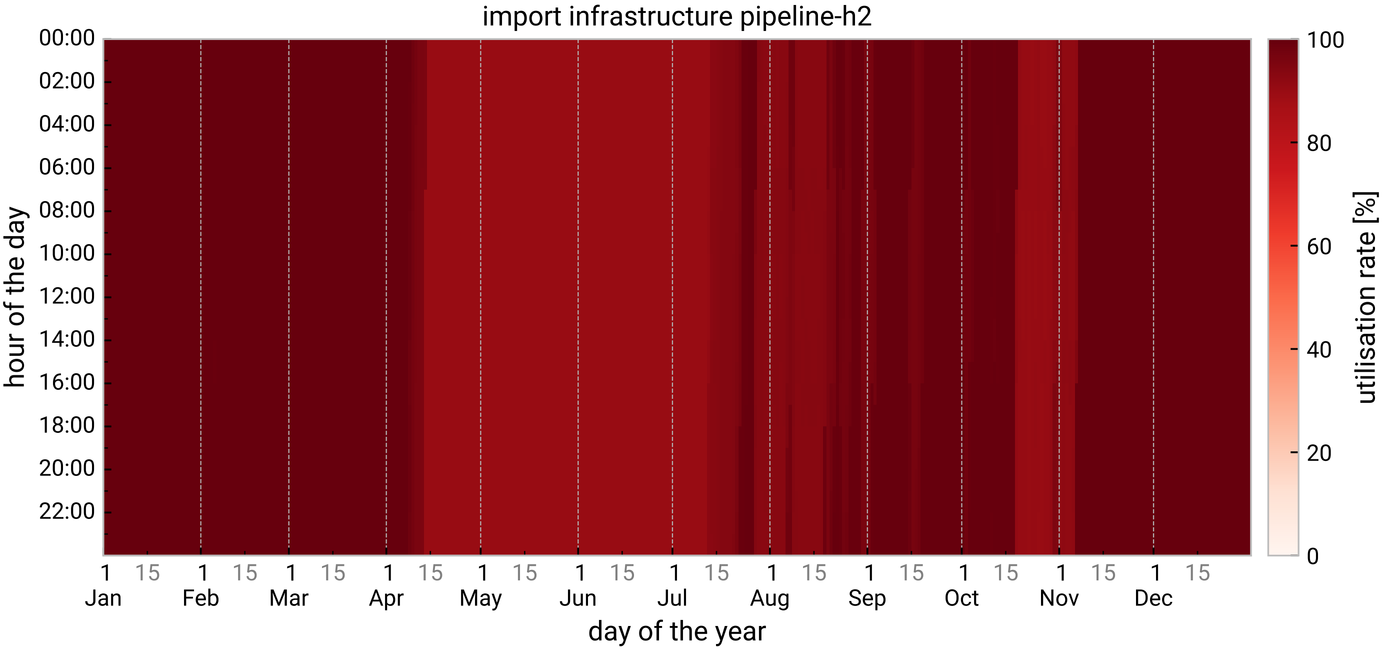


Supplementary Figure 30: **Temporal usage pattern of electricity and hydrogen storage.** The capacity-weighted average utilisation rate is 75+% for import HVDC links (**a**) and 90+% for hydrogen pipelines (**b**). For hydrogen import pipelines, a clear seasonal pattern with higher utilisation in winter is visible. For other energy or material imports than hydrogen and electricity, the timing of imports is not informatively captured due to problem degeneracy caused by negligible storage costs of carbonaceous fuels and steel. HVDC = high-voltage direct current.

**a** state of charge of hydrogen storage

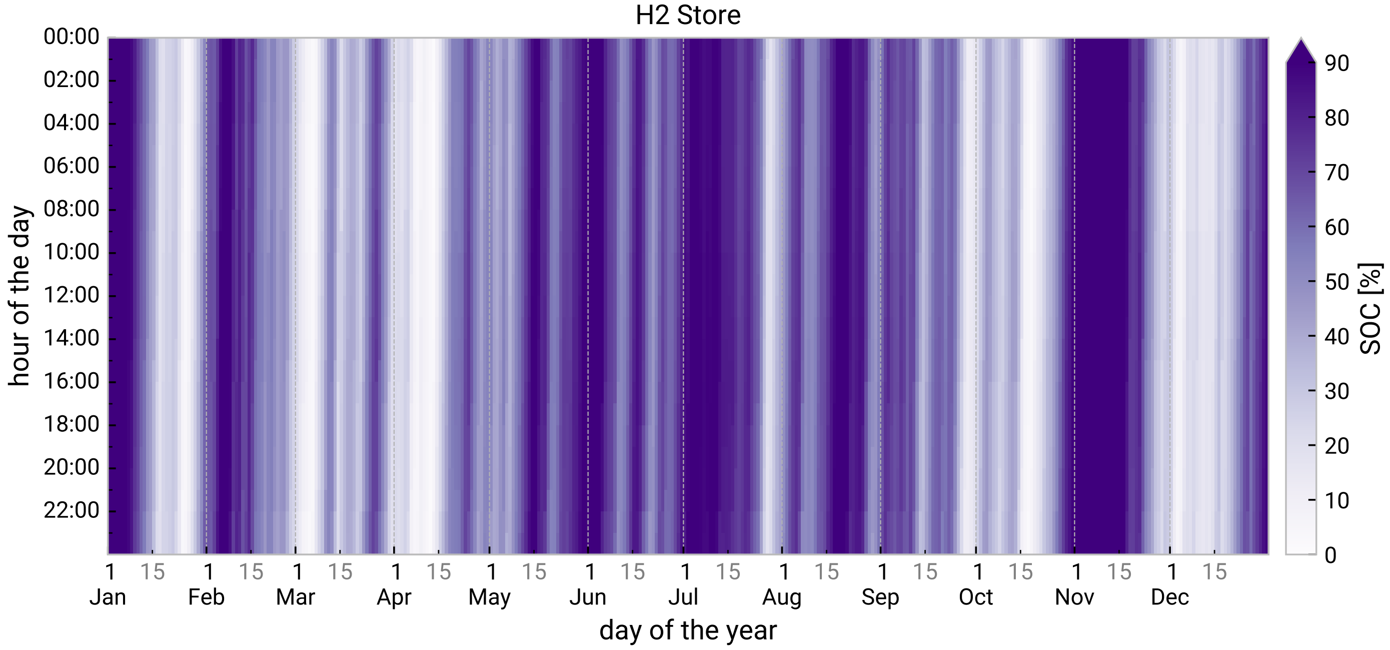


**b** state of charge profile of thermal storage in district heating

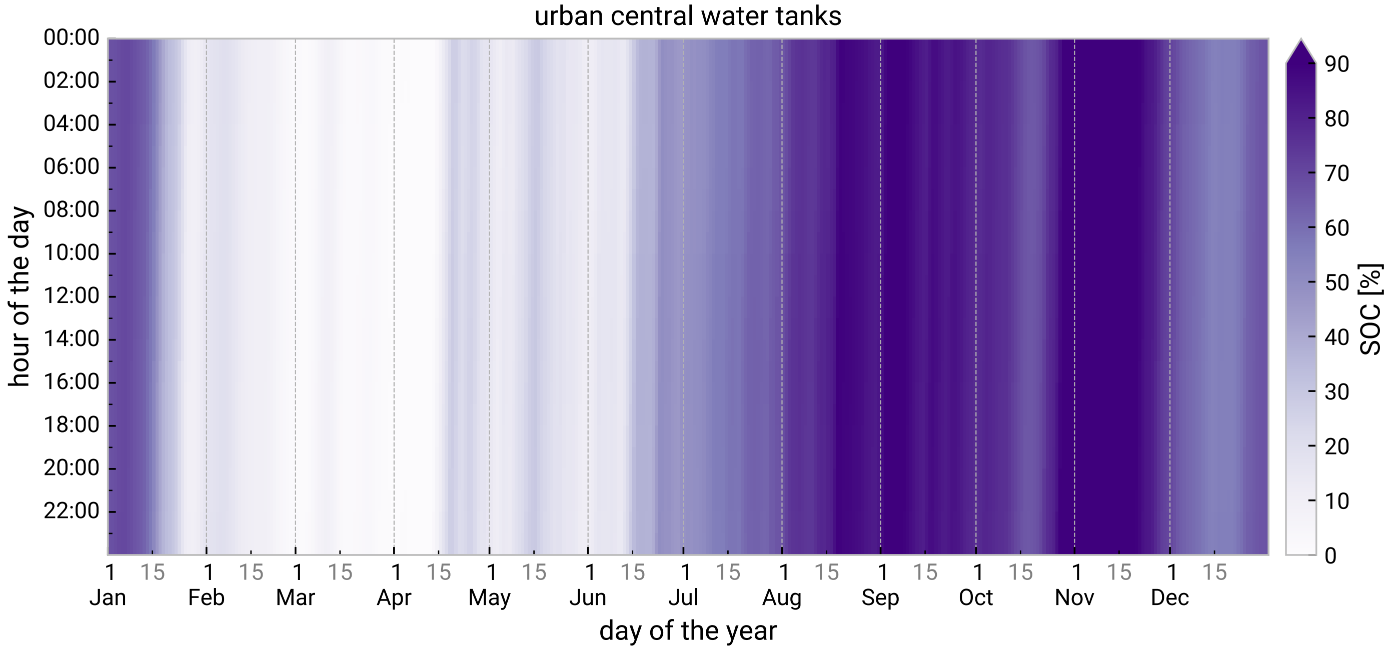


Supplementary Figure 31: **State-of-charge profile of long-duration energy storage in the domestic scenario without imports.** Hydrogen storage (**a**) is used synoptically, whereas district heating storage (**b**) is used seasonally.

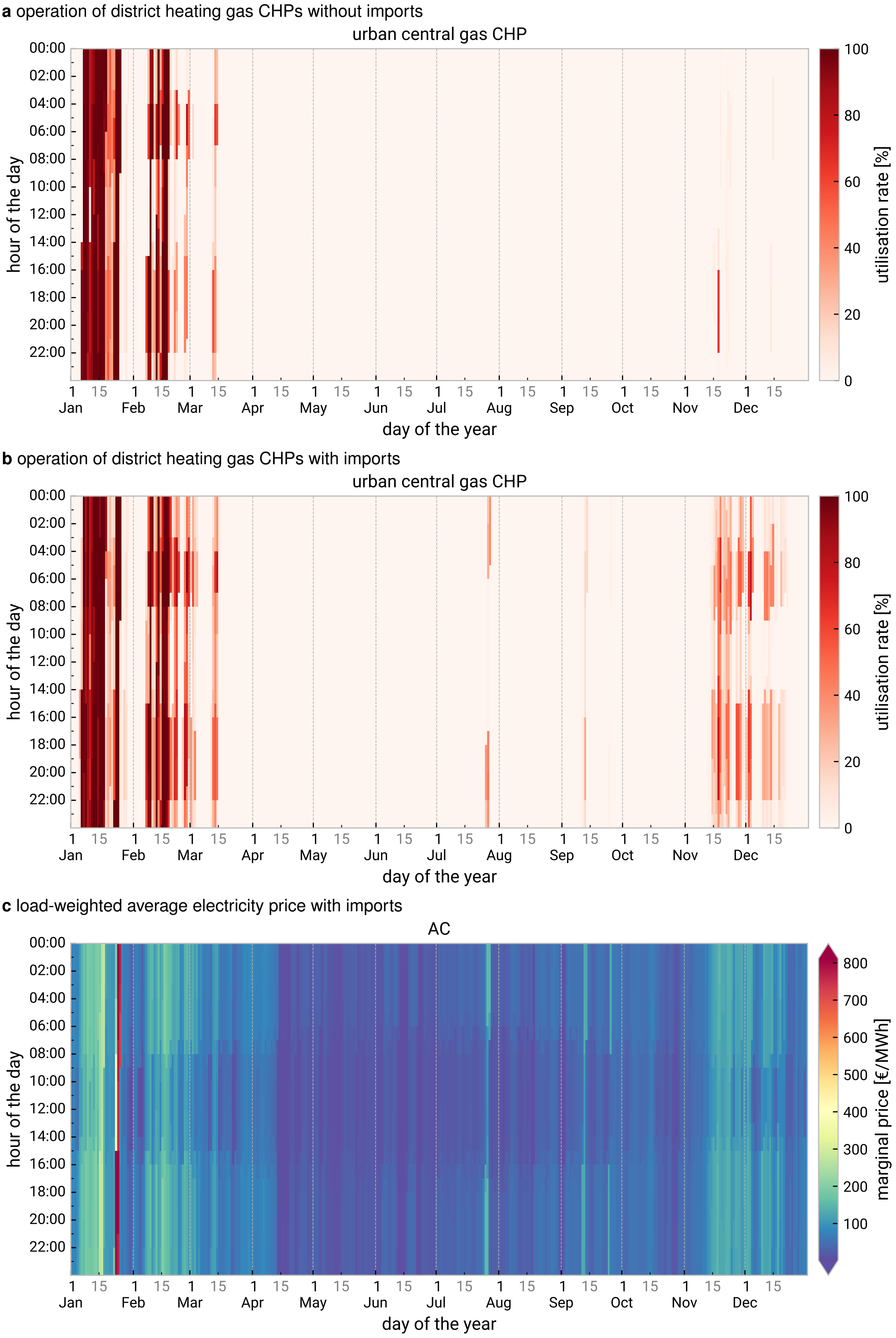


Supplementary Figure 32: **Temporal usage pattern of backup power/heat in relation to import scenario.** In both cases without imports (**a**) and with imports (**b**), gas CHPs were the main backup option, running for few days during winter when prices are higher, as the load-weighted average electricity prices in the scenario without imports show (**c**). Backup power plant operation is higher when imports displace power-to-X flexibilities. CHP = combined heat and power.

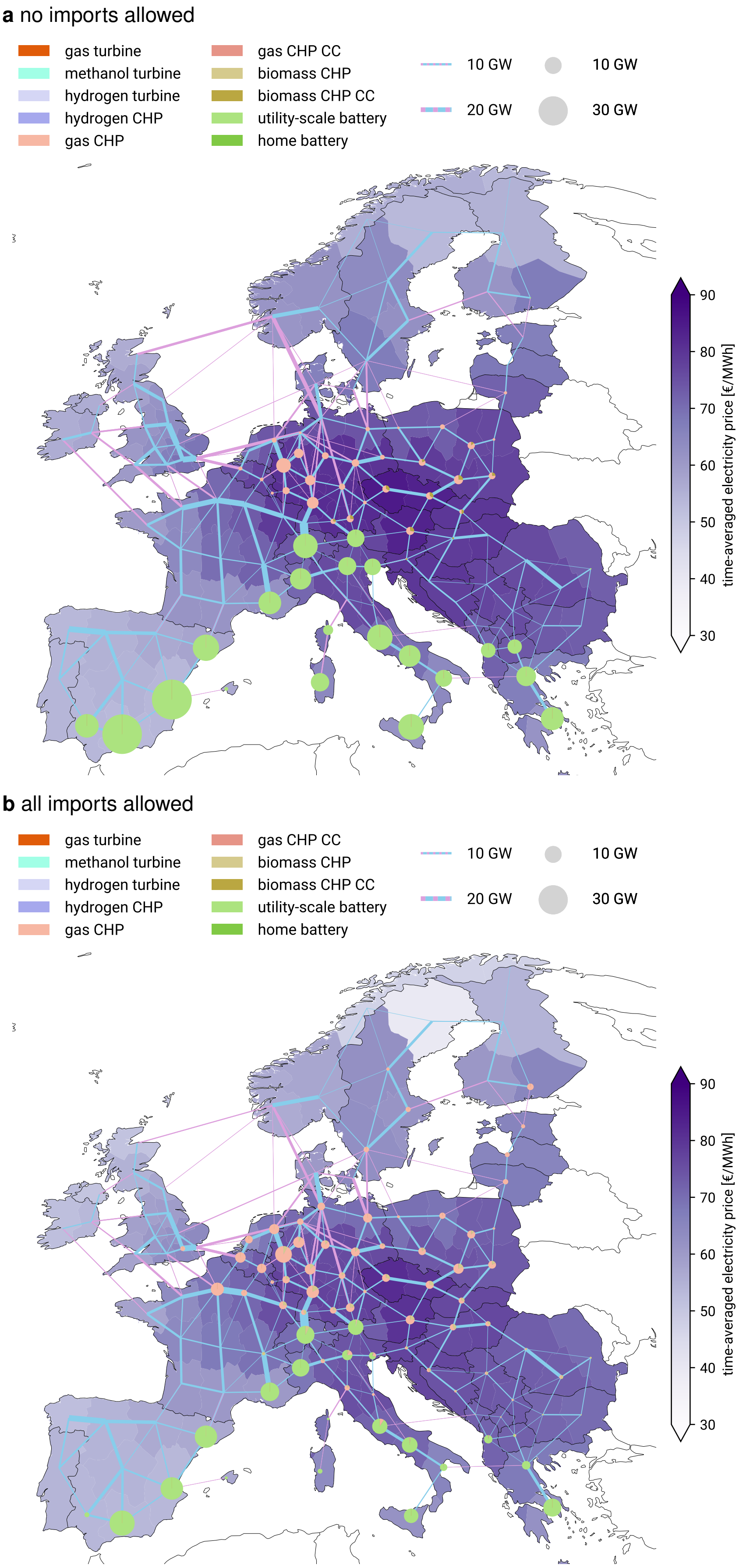


Supplementary Figure 33: **Spatial distribution of backup power.** Scenarios with no imports (**a**) and all imports (**b**) allowed. Batteries are concentrated in Southern Europe. Gas-fired combined heat and power plants are distributed across Central Europe where electricity prices are higher, with lower build-out when no imports are allowed as domestic power-to-X flexibility reduces the need for backup capacities. Instead of firing up reserve power plants, the production of power-to-X plants is curtailed. Power transmission infrastructure distributes backup capacities across Europe where there are none. Blue lines represent HVAC lines, rosa lines represent HVDC links. Maps made with Natural Earth. CHP = combined heat and power; CC = carbon capture; HVAC = high-voltage alternating current; HVDC = high-voltage direct current.

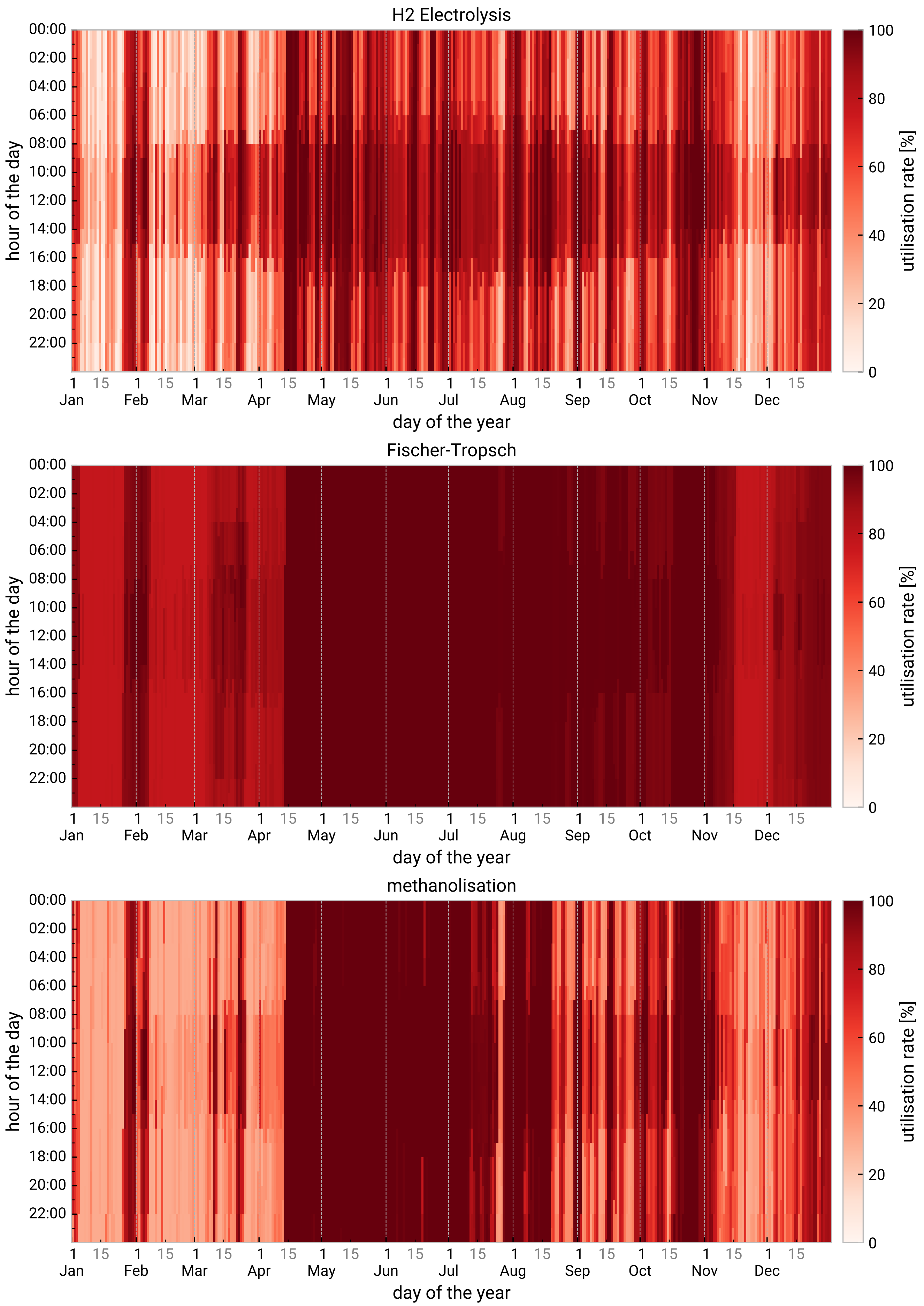


Supplementary Figure 34: **Temporal usage patterns of selected power-to-X technologies in scenario without imports.** Electrolysis clearly reacts to the availability of wind and solar electricity, despite high unit investment costs of 950€/kW$_e$. Fischer-Tropsch runs more steadily, reducing production over much of the challenging winter months to its minimum part-load of 50%. The methanolisation process can be used more flexibly with a minimum part load of 20%.

**a** without imports

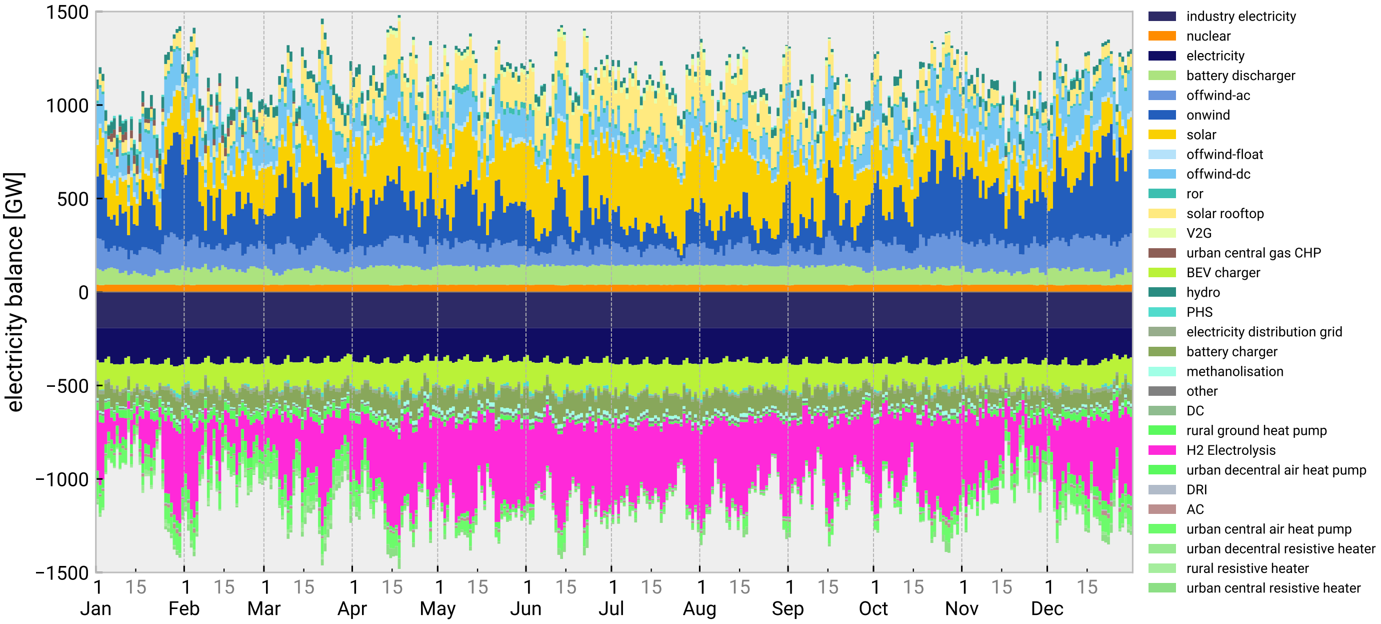


**b** with imports

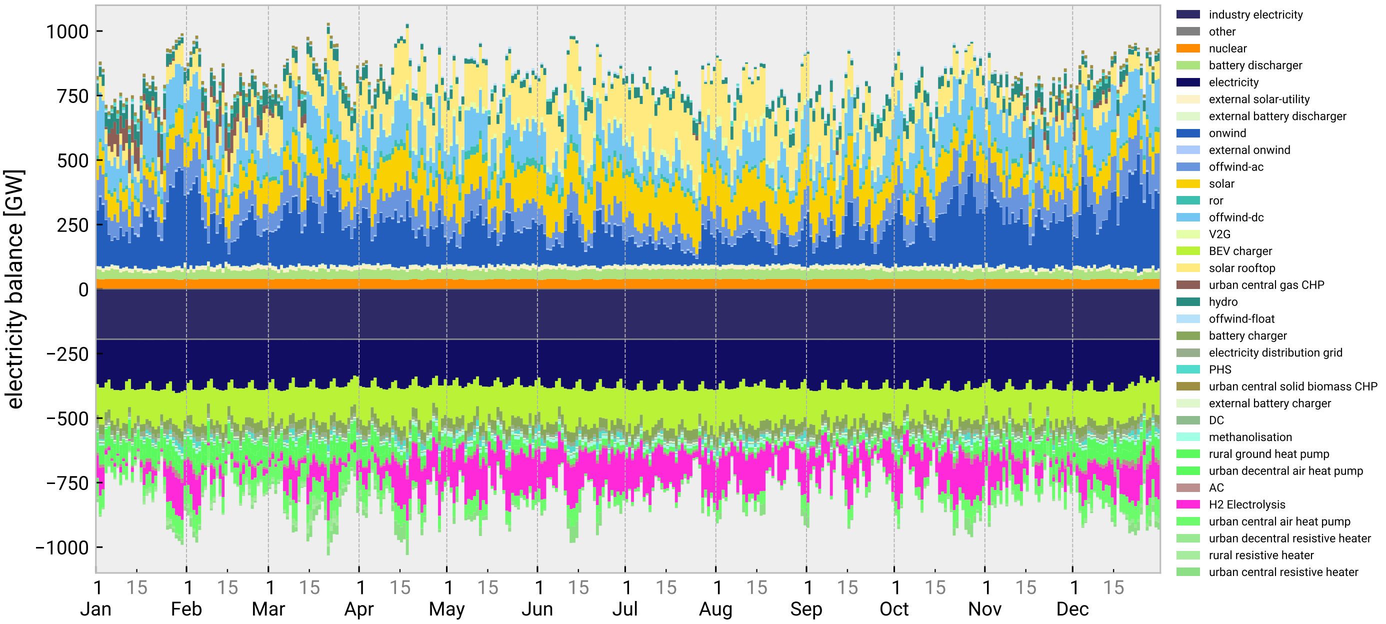


Supplementary Figure 35: **Energy balance time series for electricity.** Scenarios without imports (**a**) and with imports (**b**). Resampled to daily averages. Positive numbers indicate supply, negative numbers indicate consumption. V2G = vehicle-to-grid; CHP = combined heat and power; BEV = battery electric vehicle; PHS = pumped hydro storage; AC = alternating current; DC = direct current; DRI = direct reduced iron.

**a** without imports

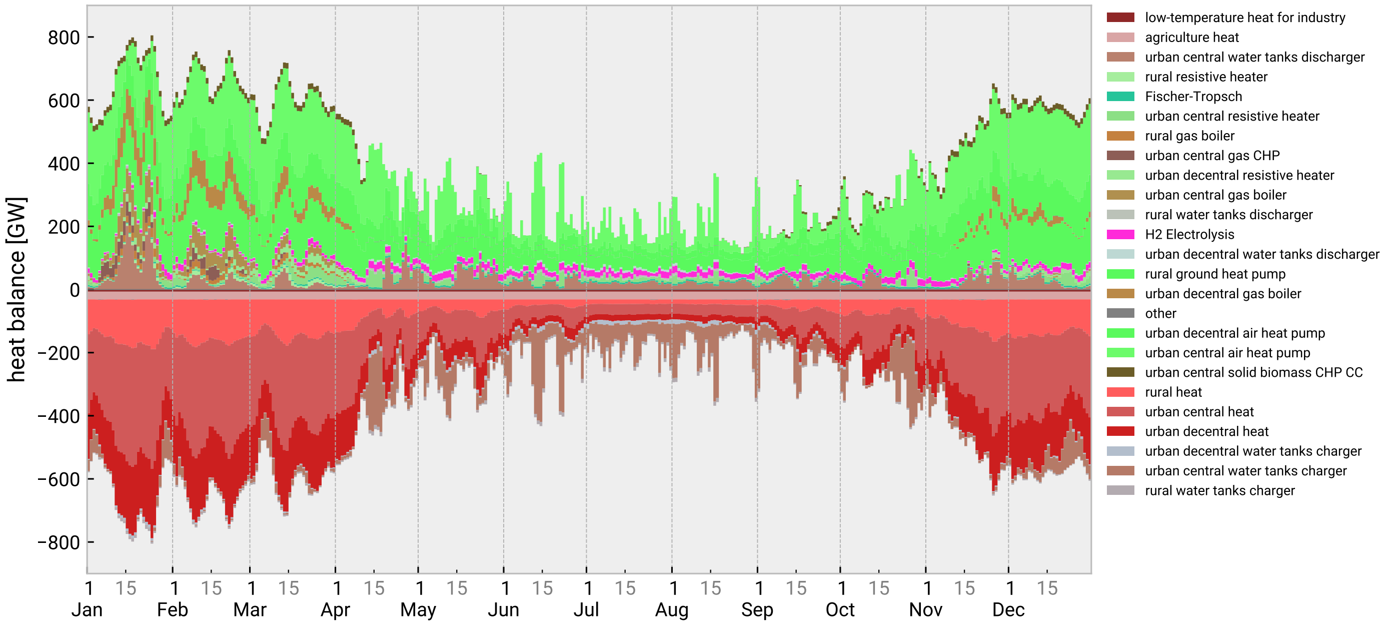


**b** with imports

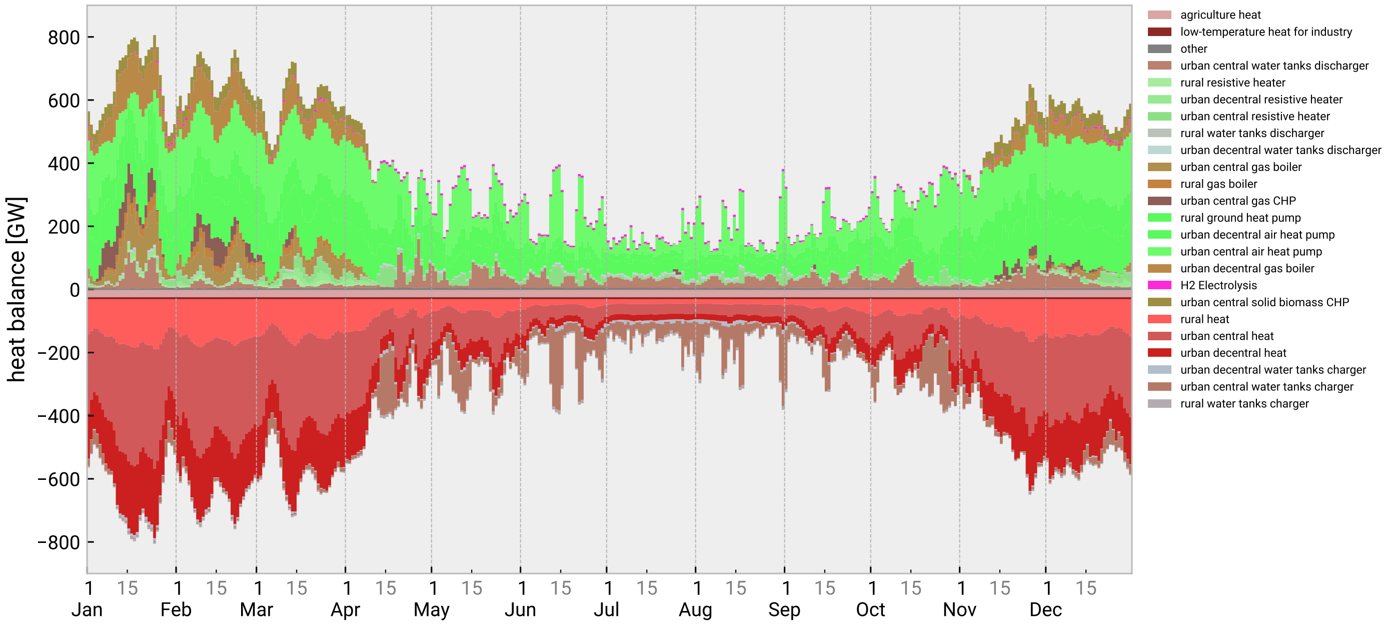


Supplementary Figure 36: **Energy balance time series for heat.** Scenarios without imports (**a**) and with imports (**b**). Resampled to daily averages. Positive numbers indicate supply, negative numbers indicate consumption. CHP = combined heat and power; CC = carbon capture.

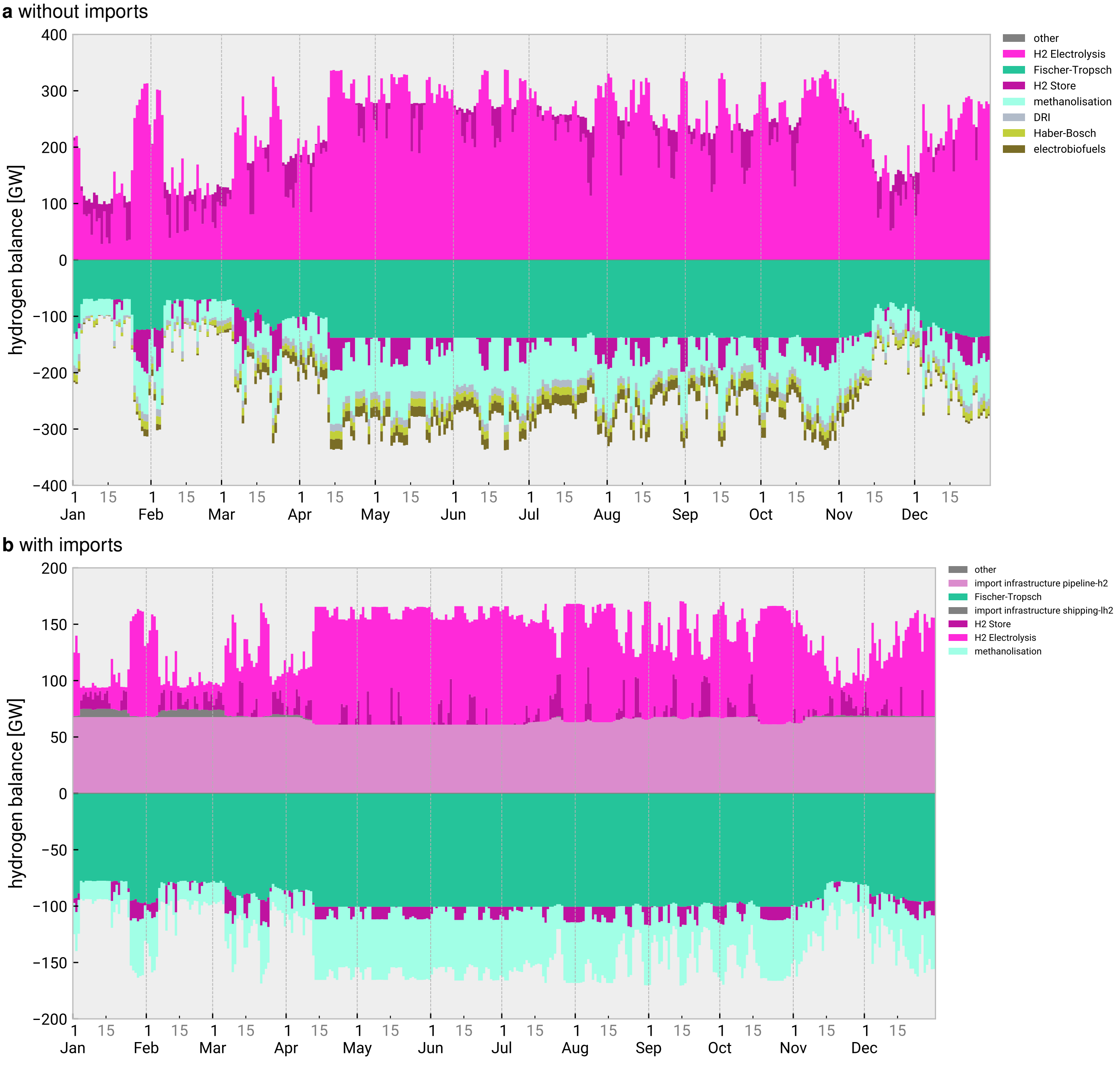


Supplementary Figure 37: **Energy balance time series for hydrogen.** Scenarios without imports (**a**) and with imports (**b**). Resampled to daily averages. Positive numbers indicate supply, negative numbers indicate consumption. DRI = direct reduced iron

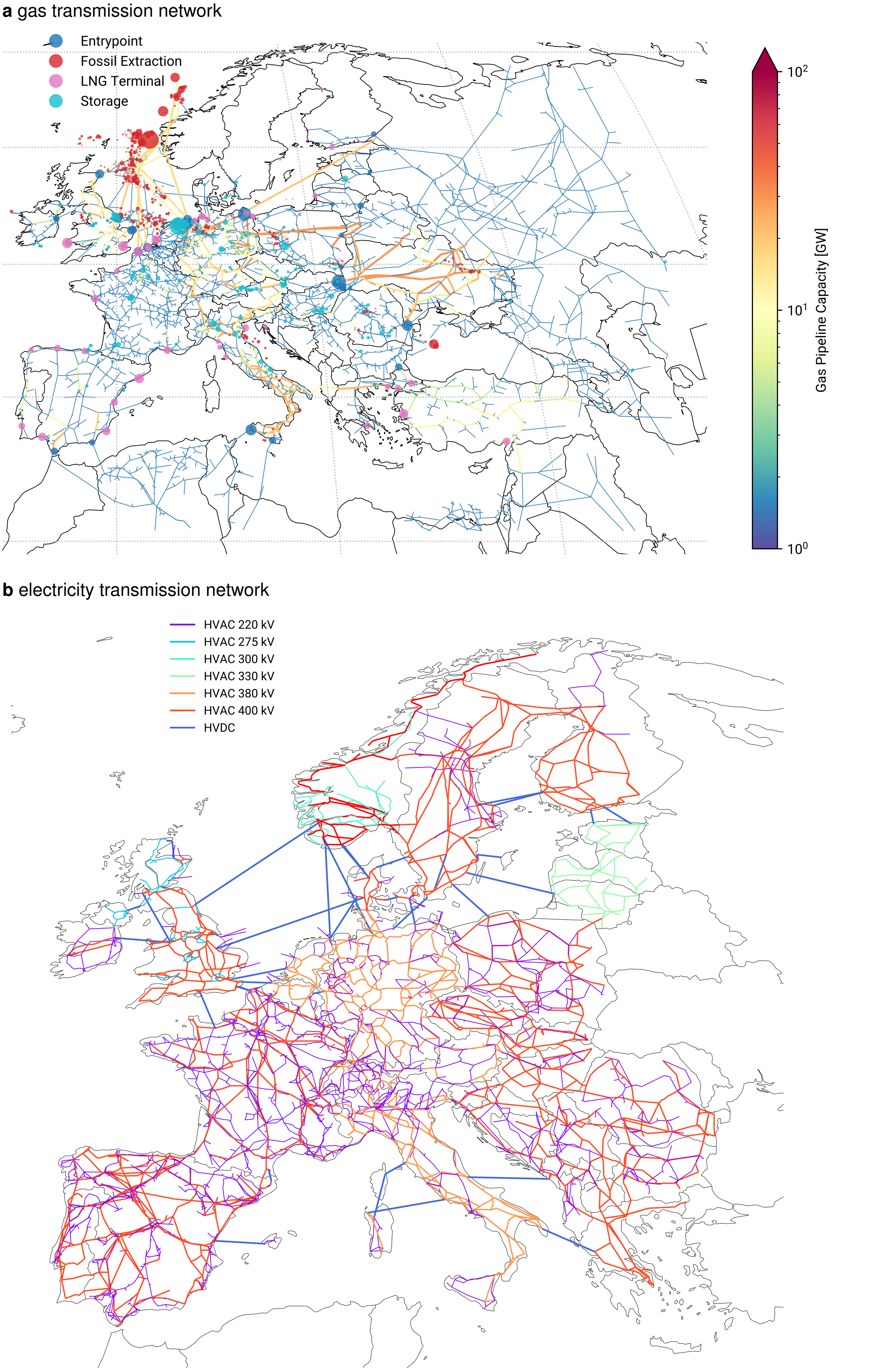


Supplementary Figure 38: **Gas and electricity transmission network data.** **a** For gas transmission, the map shows pipelines sized and colored by rated capacity, fossil gas extraction sites, storage locations, pipeline entrypoints, and LNG terminals. The data comes from SciGRID_gas and is supplemented with data from Global Energy Monitor. **b** For power transmission, the map shows existing transmission lines at and above 220 kV taken from OpenStreetMap (https://www.openstreetmap.org/), supplemented with planned TYNDP projects (https://tyndp.entsoe.eu/). Maps made with Natural Earth. LNG = liquefied natural gas; HVAC = high-voltage alternating current; HVDC = high-voltage direct current.

**a** unclustered raw data from Caglayan et al. (2020)

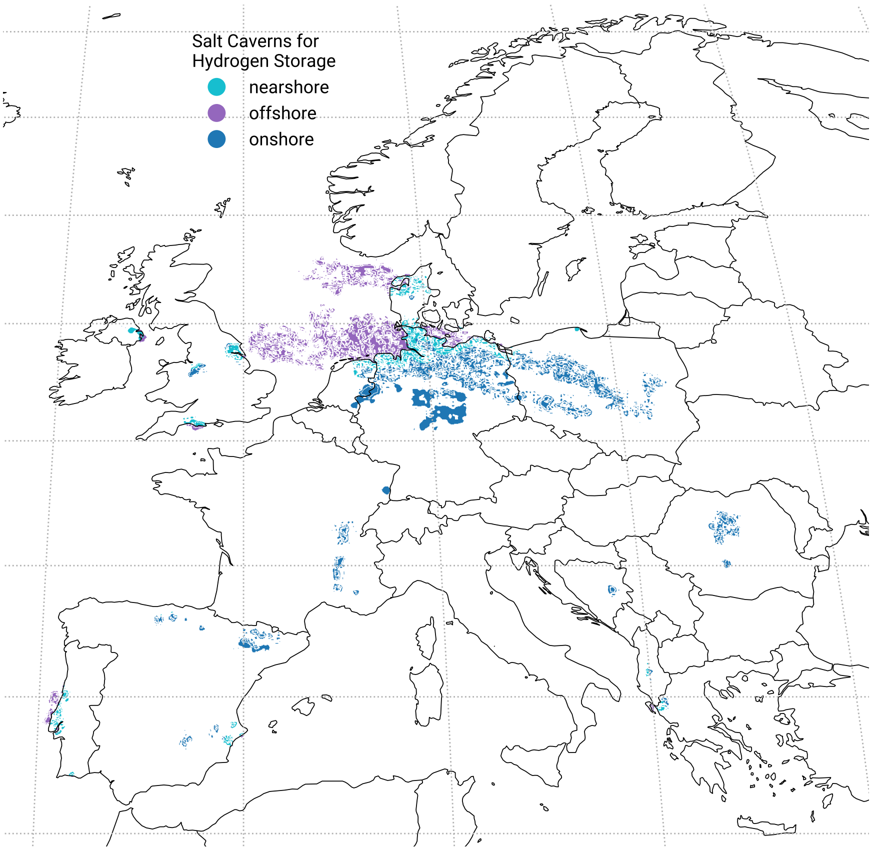


**b** clustered near-shore cavern storage potential

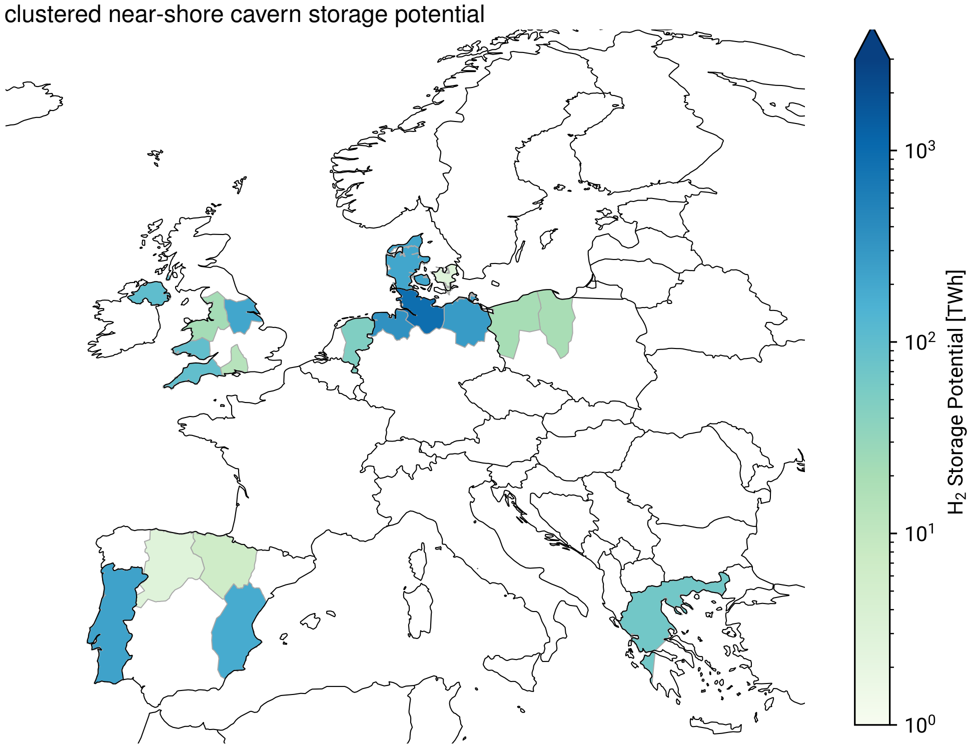


Supplementary Figure 39: **Locations considered for geological hydrogen storage in salt caverns.** Data based on Caglayan et al. [95]. Panel **a** shows unclustered raw data. Panel **b** shows clustered near-shore cavern storage potential. Only near-shore caverns are considered to minimize environmental impact of brine disposal. Maps made with Natural Earth.

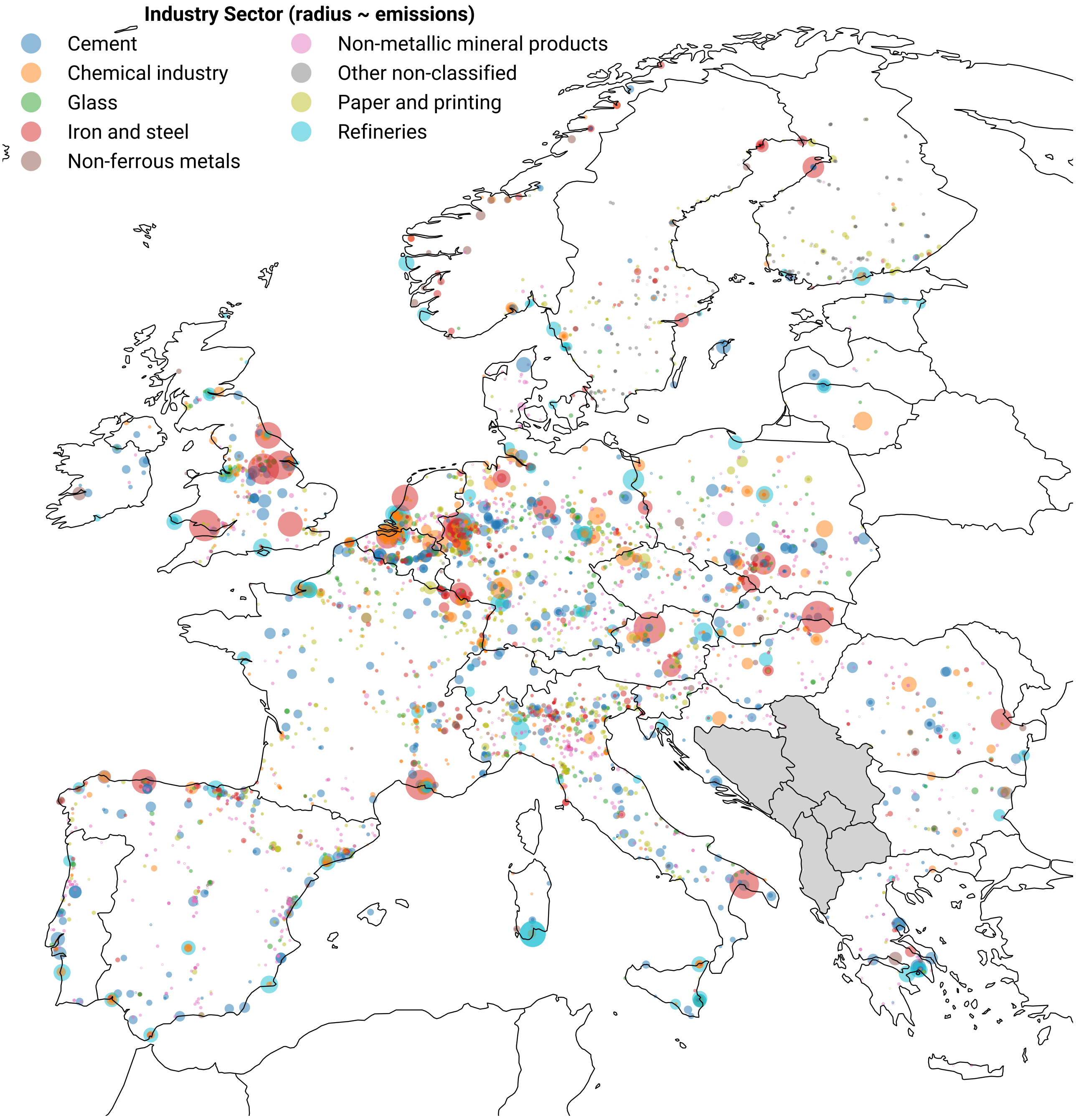


Supplementary Figure 40: **Considered locations of industrial production sites by sector.** Data based on Manz and Fleiter (2018). Marker size scales proportionally to the emissions of the respective site. This data is used for the spatial distribution of industrial energy and feedstock demands. Maps made with Natural Earth.
